# Measuring and modeling the effects of vagus nerve stimulation on heart rate and laryngeal muscles

**DOI:** 10.1186/s42234-023-00107-4

**Published:** 2023-02-17

**Authors:** William J. Huffman, Eric D. Musselman, Nicole A. Pelot, Warren M. Grill

**Affiliations:** 1 Department of Biomedical Engineering, Duke University, Fitzpatrick CIEMAS, Box 90281, Room 1427, 101 Science Drive, Durham, NC 27708-0281 USA; 2 Department of Electrical and Computer Engineering, Duke University, Durham, USA; 3 Department of Neurobiology Engineering, Duke University, Durham, USA; 4 Department of Neurosurgery Engineering, Duke University, Durham, USA

**Keywords:** Vagus nerve stimulation, Cardiac function, Laryngeal muscle, Computational models, Dynamic range

## Abstract

**Background:**

Reduced heart rate (HR) during vagus nerve stimulation (VNS) is associated with therapy for heart failure, but stimulation frequency and amplitude are limited by patient tolerance. An understanding of physiological responses to parameter adjustments would allow differential control of therapeutic and side effects. To investigate selective modulation of the physiological responses to VNS, we quantified the effects and interactions of parameter selection on two physiological outcomes: one related to therapy (reduced HR) and one related to side effects (laryngeal muscle EMG).

**Methods:**

We applied a broad range of stimulation parameters (mean pulse rates (MPR), intra-burst frequencies, and amplitudes) to the vagus nerve of anesthetized mice. We leveraged the in vivo recordings to parameterize and validate computational models of HR and laryngeal muscle activity across amplitudes and temporal patterns of VNS. We constructed a finite element model of excitation of fibers within the mouse cervical vagus nerve.

**Results:**

HR decreased with increased amplitude, increased MPR, and decreased intra-burst frequency. EMG increased with increased MPR. Preferential HR effects over laryngeal EMG effects required combined adjustments of amplitude and MPR. The model of HR responses highlighted contributions of ganglionic filtering to VNS-evoked changes in HR at high stimulation frequencies. Overlap in activation thresholds between small and large modeled fibers was consistent with the overlap in dynamic ranges of related physiological measures (HR and EMG).

**Conclusion:**

The present study provides insights into physiological responses to VNS required for informed parameter adjustment to modulate selectively therapeutic effects and side effects.

**Supplementary Information:**

The online version contains supplementary material available at 10.1186/s42234-023-00107-4.

## Introduction

Vagus nerve stimulation (VNS) for treatment of heart failure (HF) showed promise in preclinical experiments (Li et al. 2004; Sabbah et al. 2005), but subsequent clinical studies failed to meet efficacy endpoints (De Ferrari et al. 2014; Gold et al. 2016). A potential contributor to this discrepancy is the difference in stimulation titration (Ardell et al. 2017; Musselman et al. 2018): VNS in preclinical studies often produced a slowing of heart rate (HR, bradycardia), whereas clinical stimulation amplitudes were limited by patient tolerance to side effects such as throat discomfort, and thus did not produce bradycardia (Sharma et al. 2020). We conducted preclinical animal studies and computational modeling to identify stimulation parameters that were most effective at differentially modulating the effects of VNS on HR and skeletal muscle action (EMG), and the results inform parameter adjustment in future studies of VNS.

Devices using electrical stimulation should selectively activate fibers mediating therapeutic effects while minimizing activation of off-target fibers causing side effects (Fitchett et al. 2021). For example, VNS protected against ventricular fibrillation during carotid artery occlusion in dogs at stimulation intensities that produced bradycardia (Vanoli et al. 1991). The therapeutic effect was preserved during atrial pacing, indicating that treatment was associated with, but not directly mediated by, bradycardia (Li et al. 2004; Musselman et al. 2018). Proposed mechanisms of action include improved autonomic balance, suppressed inflammation in cardiomyocytes, and inhibition of the renin-angiotensin-aldosterone system (Sabbah et al. 2011). A VNS protocol may aim to stimulate fibers responsible for bradycardia while avoiding fibers that cause laryngeal muscle contraction. However, achieving this separation is complicated by the heterogenous topology of the cervical vagus nerve (CVN) and the dependency of stimulation thresholds on nerve fiber diameter (Pelot and Grill 2020; Thompson et al. 2019; Yoo et al. 2013). Large, myelinated A fibers mediate activation of muscles of the larynx and have lower stimulation thresholds while smaller, thinly myelinated B fibers produce slowing of HR and have higher stimulation thresholds (Nicolai et al. 2020; Yoo et al. 2016). There is a need for alternative approaches that mitigate the detrimental effects of concomitant activation of off-target fibers and account for the complex relationships between VNS-evoked muscle activation and HR reductions. Temporal patterns of stimulation may enable selective modulation of the physiological effects of activating target and off-target fibers (Yoo et al. 2016).

Computational models of neural stimulation are used to synthesize experimental data, prototype novel electrode designs, evaluate new stimulation parameters, and inform clinical diagnoses and treatment (Davis et al. 2007; Howell and McIntyre 2017; Lempka et al. 2020; Niederer et al. 2019). Applications of computational models to peripheral nerve stimulation are largely limited to studies of nerve fiber activation (Helmers et al. 2012; Musselman et al. 2021). However, given the widespread physiological therapeutic and side effects of VNS, there is a need for computational models of the end-organ responses to patterns of nerve fiber activation.

We paired preclinical in vivo recordings of HR and laryngeal EMG in mice with integrated computational models to explore the effects and interactions of stimulation parameter selection on nerve and physiological responses to VNS. We quantified in vivo HR and EMG responses across amplitudes, frequencies (2 − 100 Hz), and temporal patterns of stimulation (regular, burst, and random). These data were leveraged to parameterize and validate computational models of the physiological responses (HR and laryngeal muscle activation) to VNS. The models were then used to gain insight into physiological effects. These findings may be used to establish patterns of stimulation to control physiological responses and increase the dynamic range between generation of desired physiological responses and undesired side effects.

## Methods

### Animal preparation

We measured physiological responses to VNS in 10 adult C57BL/6 J mice (4 females, 6 males, 8-10 weeks of age, Jackson Labs, Bar Harbor, ME, USA). We measured responses to an expanded range of stimulation amplitudes in an additional 4 animals (all males), and 27 animals were required for protocol development and experimental troubleshooting (41 total). We reproduced the sample size used by a comparable study in dogs (Yoo et al. 2016) to power the current study, and all subjects with recorded HR and EMG were included in the analysis. Primary experimental measures were quantified HR and EMG, and the secondary calculated endpoint was effect score (see Signal quantification). The experimenter remained unblinded, and randomization of stimulation parameters and automated quantification of outcomes accounted for unblinded data collection.

Animals were anesthetized in an induction chamber with 5% sevoflurane and moved to a heating pad for the duration of the surgery where rectal temperature was monitored continuously and maintained approximately at 37 °C. We delivered maintenance anesthesia through a nose cone with sevoflurane (1.5-3%) and monitored anesthesia depth by heart rate and toe pinch reflex. We made a ventral midline incision to access the cervical space and isolated the right CVN from the carotid artery and internal jugular vein using blunt dissection. We implanted a bipolar cuff electrode on the CVN with 200 μm inner diameter and 895 μm center-to-center contact spacing (FNC-200-V-R-A-30, Micro-Leads, Somerville, MA, USA). There was no explicit control of electrode polarity and different animals received a mix of distal cathode-leading and anode-leading bipolar stimulation.

#### Signal collection

**Fig. 1:**
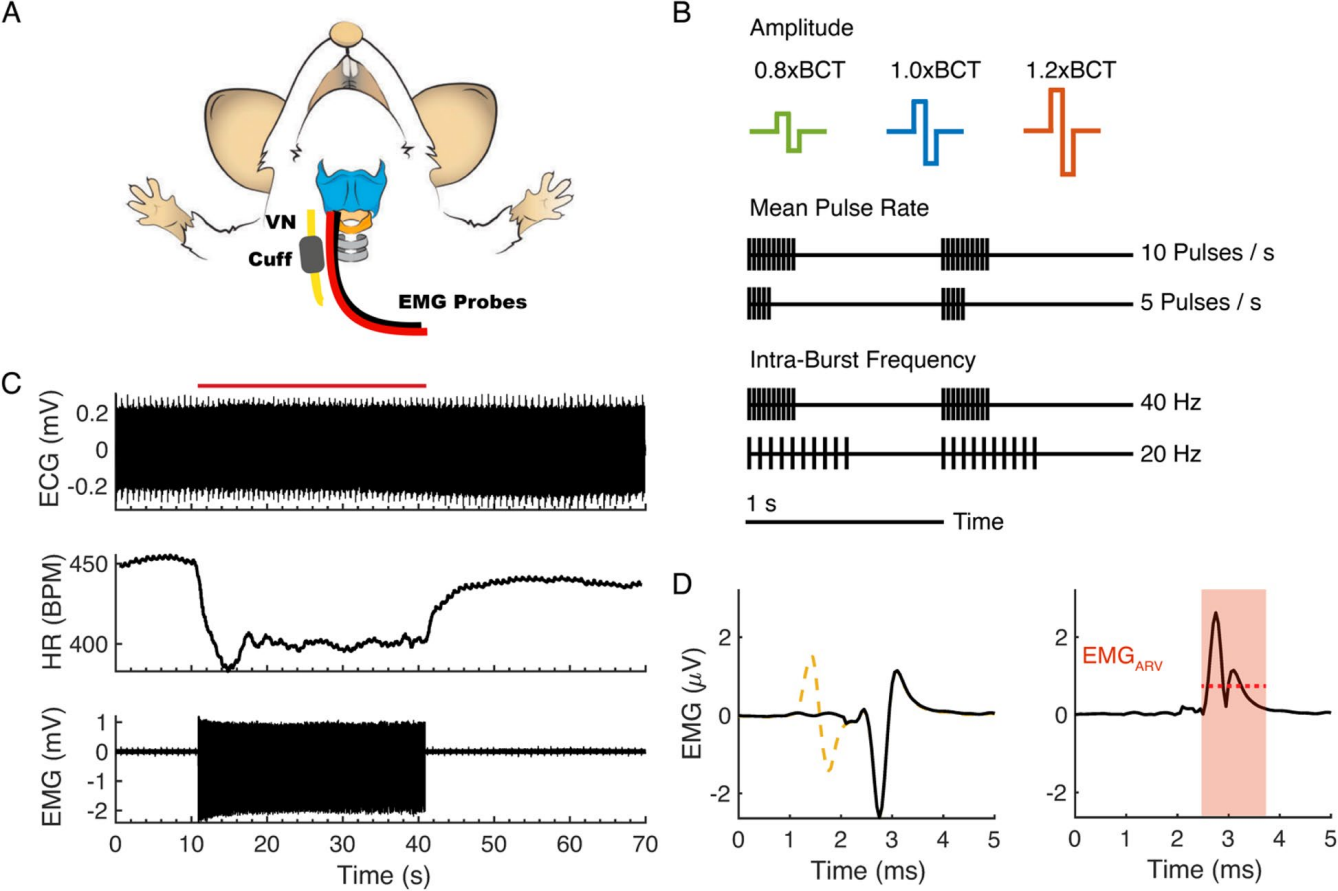
In vivo data acquisition, stimulation parameter design, and outcomes quantification. **A** Schema of vagus nerve stimulation (VNS) showing cuff implantation site on the right cervical vagus nerve and two wire electrodes placed under the thyroid cartilage to measure EMG (adapted from (Tabler et al. 2017) under the Creative Commons Attribution License). **B** VNS parameters. “Amplitude”: stimulation amplitude of symmetric biphasic pulse (300 μs per phase) normalized to bradycardia threshold (BCT). “Mean Pulse Rate”: number of pulses applied per burst for 1 s repeating patterns (e.g., 10 pulses/s, 5 pulses/s). “Frequency”: intra-burst frequency of pulses (e.g., 40 Hz, 20 Hz). **C** Illustrative data from single trial (1.0xBCT, 20 Hz constant frequency). ECG (top) used to quantify heart rate (HR, middle). EMG collected from same trial (bottom). VNS begins at t = 10 s and ends at t = 40 s (red bar). **D** Stimulation artifact removal using template subtraction and quantification of VNS-evoked EMG. Template subtraction was performed to remove stimulus artifact (left). Stimulus was applied at 1 ms and trigger signal was used to identify time-course of pulse (yellow dashed line at left). Second-wave (starting at 2.5 ms) was identified as VNS-evoked EMG. Rectified EMG quantified using average rectified value (EMG_ARV_, red dashed) during window of EMG waveform (right, red shading)

Stimulation and recording were controlled using custom software in MATLAB R2016a (MathWorks, Natick, MA, USA) and LabChart v7.3.8 (ADInstruments, Sydney, Australia). All signals were collected using a PowerLab/16SP (ADInstruments). We recorded ECG using a 3-lead configuration with the right forelimb as positive contact, left forelimb as the negative contact, and the left hindlimb as ground. We removed the insulation from the tips of 2 stainless steel wires (0.0054-in. diameter) and placed them under the right side of the thyroid cartilage to measure EMG signals from laryngeal muscles (Fig. 1A). ECG and EMG signals were amplified with 400x gain by a preamplifier (C-ISO-256, iWorx, Dover, NH, USA) and 10x by a biopotential amplifier (ETH-256, iWorx), band-pass filtered between 10 Hz and 1 kHz, and sampled at 5 kHz.

Stimulation voltage was produced by a Powerlab/16SP and converted to current using an analog stimulus isolator with a 1 V-to-1 mA conversion factor (Model 2200, A-M Systems, Sequim, WA, USA). We delivered symmetric, biphasic, charge-balanced pulses with 300 μs per phase. Amplitude was titrated to produce a ~ 10% reduction in HR in response to 20 Hz VNS, which we defined as bradycardia threshold (BCT). We reassessed BCT every 10 trials.

We applied constant frequency VNS between 2 and 100 Hz and designed burst patterns of stimulation to quantify the effects of intra-burst frequency and mean pulse rate (MPR) (Fig. 1B). Clinically, VNS is typically applied at 20-30 Hz (Musselman et al. 2018; Thompson et al. 2021). Patterns were repeated in concatenated 1 s epochs and MPR was calculated by the number of pulses applied per second; thus, the MPR is equivalent to the frequency for constant frequency stimulation and to the number of pulses per burst for burst pattern stimulation. We evaluated 8 constant frequencies and 28 burst patterns (36 temporal patterns total), with intra-burst frequencies between 2 and 100 Hz and MPR between 2 and 100 pulses/s, which corresponded to 2 to 100 pulses per burst.

Stimulation frequencies and burst patterns were applied at BCT (1.0xBCT), 20% below BCT (0.8xBCT), and 20% above BCT (1.2xBCT) in randomized block trial format (108 combinations total). Pattern order was generated with the randperm( ) function in MATLAB and experiment subject order was alternated based on sex. Each trial began with 10 s without stimulation to assess baseline, 30 s of VNS, and 30 s without stimulation for recovery (Fig. 1C).

We tested random patterns of VNS and their constant frequency controls at the end of the experiment. We designed random patterns of stimulation at 10 Hz and 20 Hz MPR and compared outcomes to constant frequency stimulation at 10 Hz and 20 Hz, respectively. Random patterns were novel for each animal and produced by randomizing stimulation pulse positions within a 1 s epoch and repeating the pattern for 30 s. Patterns were not designed with uniform burst frequency or inter-pulse interval. Mean inter-pulse frequency ranged between 13.0 and 35.6 Hz for random patterns with 10 Hz MPR and between 32.4 and 75.4 Hz for random patterns with 20 Hz MPR. For each random stimulation pattern, we performed a trial with the constant frequency at the chosen MPR (C_1_), followed by a trial of a random pattern of stimulation (R), and ended with another trial of constant frequency (C_2_).

We performed an end-of-study vagotomy (VNX) in all animals distal to the cuff to determine whether the observed responses were mediated by stimulation of afferent fibers within the cuff, efferent fibers within the cuff, or current leakage. To quantify the effects of VNX, we performed a trial (1.0xBCT, 20 Hz constant frequency) before and after vagotomy, and we quantified the resulting changes in EMG and HR.

In one animal, we only tested a single stimulation amplitude (animal HU0: 1.0xBCT). High noise in the electrophysiological signal led to the removal of five trials in two animals (Animal NN5: 1.2xBCT, 50 Hz intra-burst frequency, 10 pulses/s; 1.0xBCT, 100 Hz intra-burst frequency, 30 pulses/s. Animal YR5: 0.8xBCT, 50 Hz intra-burst frequency, 20 pulses/s; 1.2xBCT 100 Hz intra-burst frequency, 20 pulses/s; 1.0xBCT, 100 Hz intra-burst frequency, 30 pulses/s). In one animal from the temporal pattern experiments described above (animal DP8) and in four additional animals (animals AA1, AB1, LV4, and WI7), we evaluated a wider range of stimulation amplitudes (0.2xBCT-2.0xBCT in steps of 0.2xBCT in randomized order) with constant frequency stimulation at 20 Hz.

#### Signal quantification

We analyzed HR offline from ECG data by quantifying the inter-QRS periods. Custom software automatically identified the positive peak of the QRS complex and calculated instantaneous HR using a sliding window (window size = 0.5 s; time increments of 0.1 ms). Baseline HR (HR_baseline_) was calculated as the mean of the instantaneous HR during the first 10 s of the trial. Stimulation was delivered for 30 s (from t = 10 to 40 s); HR during stimulation (HR_stim_) was quantified between t = 15 and 40 s, where the initial 5 s were not included to account for the transient response. We normalized HR during stimulation to pre-stimulation baseline (HR_norm_) to adjust for the variation of resting HR (typically 400 to 500 BPM):

$${\textrm{HR}}_{\textrm{norm}}={\textrm{HR}}_{\textrm{stim}}/{\textrm{HR}}_{\textrm{baseline}}$$

HR_norm_ values less than 1 indicated a reduction in HR during stimulation, i.e., bradycardia.

We removed stimulus artifact from EMG by template subtraction to visualize EMG waveform (Fig. 1D). Templates were the average signal between 1 ms before and 1 ms after stimulus trigger. We quantified VNS-evoked EMG by taking the average rectified value (EMG_arv_) during a window after each pulse based on the waveform of the evoked EMG signal (from 1 ms post-pulse to 5 to 8 ms post-pulse, depending on EMG waveform). The EMG_arv_ was then summed for all stimulation pulses delivered during the trial and normalized to the response at 1.0xBCT, 20 Hz constant frequency stimulation for that animal:

$${\textrm{EMG}}_{\textrm{norm}}={\sum}_{\textrm{i}}^{\textrm{n}}{\textrm{EMG}}_{\textrm{arv},\textrm{i}}/{\left[{\sum}_{\textrm{i}}^{\textrm{n}}{\textrm{EMG}}_{\textrm{arv},\textrm{i}}\right]}_{1.0\textrm{xBCT},20\textrm{Hz}}$$

where i is the pulse index, and n is the total number of pulses during the trial (equal to the product of MPR and stimulation time of 30 s).

To quantify the relative HR_norm_ and EMG_norm_ responses to a given stimulus, we defined an effect score that normalized both effects to the response from each animal 1.0xBCT, 20 Hz constant frequency stimulation. A positive effect score indicated a greater relative HR_norm_ response, and a negative effect score indicated a greater relative EMG_norm_ response.

$$\textrm{effect}\ \textrm{score}=\left(1-{\textrm{HR}}_{\textrm{norm}}\left)/\right(1-{\textrm{HR}}_{\text{norm},1.0\textrm{xBCT},20\textrm{Hz}}\right)-{\textrm{EMG}}_{\textrm{norm}}$$

### Computational models

We simulated VNS-evoked changes in HR and laryngeal EMG using computational models that we parameterized using our in vivo data.

#### Computational model of parasympathetic innervation of the sinoatrial node

Vagal efferents innervate post-ganglionic ICNS cells, which in turn release acetylcholine (ACh) to the sinoatrial node (SAN) and inhibit oscillatory firing of pacemaker cells (Hanna et al. 2021; Jänig 2011). To model VNS effects on HR, we designed and implemented a model of VNS-evoked vagal cardiac efferent signals through the intrinsic cardiac nervous system (ICNS) (Fig. 7A). We modeled the SAN as a network of coupled pacemaker cells with firing rate assumed to be the heart rate; the SAN firing rate slowed in response to the ACh release from the ICNS. We implemented the models in Python v3.8.5 and NEURON v7.8.2 (Hines and Carnevale 1997; Van Rossum and Drake 2009).

We first modeled the connection from the vagal efferent fibers to the ICNS. In Wistar rats, each pre-ganglionic cardiac vagal efferent synapses onto one post-ganglionic cell in the ICNS (McAllen et al. 2011), and frequency-dependent filtering occurs at the VN-to-ICNS synapse (Rimmer and Harper 2006). We created a population of 100 phenomenological post-ganglionic ICNS cells that could each be activated by a single pre-synaptic (vagal) source. We fit a function to the published frequency-dependent failure rate of vagally-evoked post-ganglionic events measured in an ex vivo preparation using Wistar rats (Rimmer and Harper 2006). The probability that an action potential in the preganglionic cell causes ACh release from the ICNS cell was a function of the time since the last action potential in the ICNS cell:

$$\textrm{Success}\ \textrm{Probability}\left(\textrm{t}\right)=1.028-2.183\ast {\left(\textrm{t}-{\textrm{t}}_0\right)}^{-0.7146}$$

where t is the time of an action potential in the VN (i.e., preganglionic cell) and t_0_ is the time of the previous action potential in the ICSN cell (i.e., post-ganglionic cell) (in ms, Additional file 1: Fig. 12). With each vagal event, a random number generator (scope _ random( ) in NEURON HOC programming language) defined a value between 0 and 1; successful transmission of the VN-to-ICNS synapse occurred if the variable was less than the Success Probability. ICNS cells in rats exhibit intrinsic firing that is linked to respiration (McAllen et al. 2011) and approximately half of the ICNS cells from that study (6 of 10) fired independently of applied stimuli. We extracted phasic firing rates from (McAllen et al. 2011) and incorporated probabilistic intrinsic firing into 50 of our 100 ICNS cells (Additional file 1: Fig. 13). Firing rates varied from 0 to 0.6 events per 50 ms bin.

We then implemented a multi-compartment model of ACh release from ICNS cells to the SAN (Dokos et al. 1996b) and validated our implementation using data from the publication (Additional file 1: Fig. 14). The three model compartments are the pre-synaptic main store (of post-ganglionic cell), the neuroeffector junction, and the extra-junctional space. Parameters were unchanged from the published model except the available pre-synaptic ACh concentration was increased from 0.04 mM to 0.075 mM to produce small fluctuations in SAN network firing rate (~ 0.2%) in response to ICNS intrinsic firing and subsequent ACh release.

Recent models of SAN cells include two oscillatory systems: the membrane clock (cell depolarization arising from transmembrane ion currents) and calcium clock (cell depolarization arising from internal calcium ion dynamics) (Maltsev et al. 2014). We used a published model of a SAN cell with both membrane and calcium clock mechanisms (Kharche et al. 2011) with updated ion current mechanisms (Morotti et al. 2021) which were: long-type calcium current (I_CaL_), potassium currents (I_to_ and I_sus_), and “funny” current (I_f_). We validated our implementation by comparing the transmembrane potential, ionic currents, and intracellular ion concentrations to data from published plots and a published version in MATLAB (Additional file 1: Fig. 15) (Morotti et al. 2021).

We expanded the single-cell SAN model to a network of 100 cells (10-by-10 grid) where cells sharing an edge were connected by 6 nS gap junctions, using published SAN gap junction conductance (Verheijck et al. 2001). We incorporated post-synaptic ACh-sensitive mechanisms consisting of ACh-gated potassium channel and modulation of the “funny” current, long-lasting calcium ion channel, and the rate of sarcoplasmic reticulum uptake of calcium (Severi et al. 2012). To account for the cell size differences between the rabbit and mouse SAN models, we scaled the maximum conductance of the ACh-gated potassium channel based on cell capacitance (original values: 0.00864 μS and 32 pF; adjusted values: 0.00675 μS and 25 pF). As the SAN is the site of initiation for propagating potentials throughout the heart, the firing rate of the SAN cells on the periphery of the network was interpreted as HR, which had a mean of 389 BPM in the absence of VNS.

We simulated VNS amplitude by altering how many of the 100 SAN cells received VNS-evoked ACh release, termed ACh density, between 0 to 100. We minimized the error between the modeled HR_norm_ and the in vivo HR_norm_ at constant frequencies between 2 and 100 Hz using a bisection search method for each stimulation amplitude. Thus, higher amplitudes were represented by higher ACh synapse densities (Table 1). Using the parameterized model, we simulated the changes in HR in response to constant frequency and burst patterns of stimulation across stimulation amplitudes, using the trial structure described for the in vivo experiments. Each simulation was run 10 times, and variability in model outcomes arose from random number generation for the success probability function at the VN-to-ICNS synapse, ICNS intrinsic firing, and positional assignment of SAN cells receiving VNS-evoked ACh.

**Table 1:** Number of the 100 sinoatrial node cells that received VNS-evoked ACh release (i.e., ACh density) across stimulation amplitudes (0.8-, 1.0-, and 1.2-times bradycardia threshold (BCT)) in a computational model of parasympathetic innervation of the sinoatrial node

Stimulation Amplitude	Simulated ACh Density
0.8xBCT	28
1.0xBCT	50
1.2xBCT	57

#### Computational model of vagal motor innervation of the larynx

We modeled VNS-mediated activation of laryngeal muscles using a published model of skeletal muscle activation and fatigue (Ding et al. 2003). The model includes two primary features: (1) force production arising from motor unit activation and (2) fatigue from persistent activation. We validated our implementation by replicating published plots of force production and dynamic fatigue parameters during a simulation of burst pulse electrical stimulation (30 Hz, 1.5 s on, 0.5 s off) (Additional file 1: Fig. 16) (Ding et al. 2003).

Computational model outcomes were calculated by the force-time integral (Fig. 8A) normalized to the 20 Hz constant frequency response (Force_norm_):

$${\textrm{Force}}_{\textrm{norm}}={\int}_{\textrm{t}=0}^{\textrm{end}}\left(\textrm{Force}\right)\textrm{dt}/{\left[{\int}_{\textrm{t}=0}^{\textrm{end}}\left(\textrm{Force}\right)\textrm{dt}\right]}_{20\ \textrm{Hz}}$$

The computational model of force had no stochasticity and was run once for each pattern of stimulation.

The relationship between EMG and force is approximately linear during isometric contractions (Ibitoye et al. 2014). The EMG-force relationship has been established for surface EMG recordings from humans during voluntary and stimulation-evoked contractions (Lawrence and De Luca 1983; Mizrahi et al. 1997). Rat laryngeal muscles are composed primarily of fast twitch motor units (Hoh 2005), and we assumed VNS-evoked contractions were isometric due to anatomical size of mouse larynx (~ 1.5 mm in diameter (Tabler et al. 2017)). Thus, we assumed a 1:1 linear relationship between in vivo EMG and modeled force (EMG_norm_ = Force_norm_).

We parameterized the model with our in vivo data using a particle swarm optimization (PSO) algorithm; the PSO method for global optimization was chosen due to its suitability for problems with multiple free parameters (8 parameters; Table 2) and continuous values of the search space. A PSO algorithm works by iteratively testing a collection (swarm) of parameter sets. The performance of each parameter set (particle) was evaluated using a cost function. The particle’s parameter values (position) were updated based on the particle’s performance and the performance of the other particles until the optimization reached a predefined iteration limit (Parsopoulos and Vrahatis 2002).

**Table 2:** Parameter units, definitions, and values for force fatigue model (Ding et al. 2003). Parameter range values were defined by values used in the referenced paper and an associated follow-up study for simulations of skeletal muscles (Doll et al. 2017). PSO-identified values were used for all simulations unless otherwise stated. Ding et al. 2003 values used in model perturbation simulations

Free parameters for muscle activation model (Ding et al. 2003)			Range minimum	Range maximum	PSO-identified value	Ding et al. 2003 value
Parameter	Unit	Definition				
A	N/ms	Scaling factor for force and shortening velocity of muscle	0.11	10	10	3.0009
K_m_	–	Sensitivity of strongly bound cross-bridges to Ca^2+^-troponin complex	0.01	0.50	0.50	0.103
τ_1_	ms	Time constant of force decline at the absence of strongly bound cross-bridges	2.00	200	2.00	50.957
τ_2_	ms	Time constant of force decline due to the extra friction between actin and myosin resulting from the presence of cross-bridges	5	100	100	100
α_A_	ms^−2^	Fatigue coefficient for force-model parameter A	1	-1	−0.51	−4.0*10^−7^
α_Km_	ms^−1^ N^−1^	Fatigue coefficient for K_m_	-1*10^− 4^	1*10^− 4^	9.97*10^−5^	1.9*10^− 5^
α_τ1_	N^− 1^	Fatigue coefficient for τ_1_	0	1*10^−3^	160*10^−7^	2.1*10^−5^
τ_fat_	s	Time constant controlling recovery of force model parameters from fatigue	80	160	160	127

Patterns of stimulation (constant frequency and burst) were assigned randomly into “training” and “testing” groups with a 50-50 split (18 patterns per group). We assessed error for “training” patterns of stimulation (training error) during the PSO iterations, and we assessed error for “testing” patterns of stimulation (testing error) and all patterns of stimulation (total error) at the end of the PSO.

The swarm had 100 particles, where each particle consisted of a parameter set that defined a candidate model. We defined the initial swarm with random values from uniform distributions bounded by the minimum and maximum values for each parameter (Table 2). For each particle, we simulated the model response to each training pattern of stimulation and calculated the corresponding cost:

$$\textrm{Cost}=\sqrt{\frac{\sum_{\textrm{n}}{\left({\textrm{Force}}_{\textrm{norm}}-\overline{{\textrm{EMG}}_{\textrm{norm}}}\right)}^2}{\textrm{N}}}$$

where n is the training pattern index, N is the total number of training patterns (18 patterns), and $\overline{{\textrm{EMG}}_{\textrm{norm}}}$ is the mean normalized EMG across animals.

The swarm was divided into 20 neighborhoods with 5 particles each; the same neighborhood groupings were maintained for the duration of the PSO run. The position of each particle was updated at the end of each iteration based on its performance, the performance of the other particles in its neighborhood, and the performance of all particles in the swarm:

$${\textrm{P}}_{\textrm{i},\textrm{new}}={\textrm{P}}_{\textrm{i},\textrm{Previous}}+{\textrm{V}}_{\textrm{i}}$$

where P_i, new_ is the updated particle position (vector of 8 parameter values), P_i, Previous_ is the current particle position, and V_i_ is the change in position (velocity) defined by:

$${\textrm{V}}_{\textrm{i}}=\text{rand}\left(\ \right)\ast \left({\textrm{P}}_{\textrm{best},1}-{\textrm{P}}_{\textrm{i}}\right)-\operatorname{rand}\left(\ \right)\ast \left({\textrm{P}}_{\textrm{best},2}-{\textrm{P}}_{\textrm{i}}\right)$$

where rand( ) is a random number drawn from a uniform distribution from 0 to 1.496 (Clerc and Kennedy 2002; Poli et al. 2007). P_best, 1_ and P_best, 2_ are defined by the performance of P_i_ relative to the ranked performance of particles within the neighborhood:

$${\displaystyle \begin{array}{ccc}\textrm{if}\ {\textrm{P}}_{\textrm{i}}={\textrm{P}}_{\textrm{best},\textrm{neighborhood}}& \to & \left\{\begin{array}{c}{\textrm{P}}_{\textrm{best},1}={\textrm{P}}_{\textrm{best},\textrm{global}}\\ {}{\textrm{P}}_{\textrm{best},2}={\textrm{P}}_{2\textrm{nd}\ \textrm{best},\textrm{neighborhood}}\end{array}\right.\\ {}\textrm{if}\ {\textrm{P}}_{\textrm{i}}={\textrm{P}}_{2\textrm{nd}\ \textrm{best},\textrm{neighborhood}}& \to & \left\{\begin{array}{c}{\textrm{P}}_{\textrm{best},1}={\textrm{P}}_{\textrm{best},\textrm{neighborhood}}\\ {}{\textrm{P}}_{\textrm{best},2}={\textrm{P}}_{3\textrm{rd}\ \textrm{best},\textrm{neighborhood}}\end{array}\right.\\ {}\textrm{else}& \to & \left\{\begin{array}{c}{\textrm{P}}_{\textrm{best},1}={\textrm{P}}_{\textrm{best},\textrm{neighborhood}}\\ {}{\textrm{P}}_{\textrm{best},2}={\textrm{P}}_{2\textrm{nd}\ \textrm{best},\textrm{neighborhood}}\end{array}\right.\end{array}}$$

where P_best, global_ is the lowest-cost particle in the whole swarm and P_best, neighborhood_ is the lowest-cost particle within the neighborhood of P_i_. The position of P_best, global_ was not updated. P_best, global_ could also P_best, neighborhood_ in its neighborhood.

Each PSO run consisted of 50 iterations. The particle with the best performance (lowest cost) in the final iteration was tested with the 18 combinations of stimulation patterns that were not used for training (testing error).

We performed 5 PSO runs, each with a unique initial population and with unique “training” and “testing” groups (Additional file 1: Fig. 17). The final model was selected based on the lowest total error across the 5 PSO runs (Table 2, Additional file 1: Fig. 17A), although it did not have the lowest training error. To confirm that the initial particle position did not determine the PSO parameter values, we performed 5 additional PSO runs using the same “training” and “testing” patterns of stimulation used to identify the optimal solution but with different initial particle positions; the PSO converged on the same cost function value and parameter values (Additional file 1: Fig. 17B). All PSO runs had a stable minimum cost across the swarm after the 20th iteration, indicating that 50 iterations were sufficient for convergence.

#### Computational model of fiber activation in mouse VNS

We modeled mouse VNS using ASCENT v1.1.4 (Musselman et al. 2021) to create a finite element model of the nerve and cuff electrode in COMSOL Multiphysics v5.6 (COMSOL Inc., Burlington, MA), solve for electric potentials in the tissue, and apply the potentials using the in vivo stimulation waveform to models of biophysically-realistic mammalian myelinated fibers in NEURON v7.6 (Hines and Carnevale 1997).

Figure 11D shows the geometry of the finite element model of mouse VNS. We used ASCENT’s mock nerve morphology generator to create a cross section of a generalized mouse cervical vagus nerve that was monofascicular nerve and 180 μm in diameter (Stakenborg et al. 2020). We extruded the cross section 25 mm longitudinally. We modeled the 200 μm diameter bipolar Micro-Leads cuff (Somerville, MA, USA) that was used in vivo and placed it halfway along the nerve, with 10 μm between the nerve and the cuff’s inner surface. We modeled a 10 μm thick saline layer over all surfaces of the cuff. The nerve and cuff were modeled in a cylinder of skeletal muscle (6 mm in diameter, 25 mm in length), and we grounded all outer surfaces of the model.

We assigned material conductivities to all domains using values from literature (Table 3). The perineurium was defined by a surface impedance (Pelot et al. 2019; Weerasuriya et al. 1984):

$${\textrm{R}}_{\textrm{m}}=\frac{\textrm{th}{\textrm{k}}_{\textrm{peri}}}{\upsigma_{\textrm{peri}}}$$

with thickness based on published linear fits to fascicle diameter in rat cervical vagus nerves (Pelot et al. 2020).

$$\textrm{th}{\textrm{k}}_{\textrm{peri}}=0.01292\ast {\textrm{d}}_{\textrm{fasc}}+1.367$$

where thk_peri_ and d_fasc_ are in microns. We modeled the cuff with a silicone substrate and thin platinum contacts, and we modeled a point current source in each contact (Pelot et al. 2018). We used COMSOL’s conjugate gradients to solve Laplace’s equation using second order solution and geometry shape functions for each contact delivering 1 mA. We weighted and summed the contributions of each contact to calculate extracellular potentials for bipolar stimulation. We confirmed that activation thresholds did not change by more than 2% when compared to models with higher mesh density, longer model length, or wider model diameter (Howell et al. 2014).

**Table 3:** Material conductivities used in the finite element model of mouse VNS

Material	Electrical Conductivity σ [S/m]	References
Muscle	{0.086, 0.086, 0.35}	(Gielen et al. 1984)
Silicone	10^−12^	(Callister and Rethwisch 2012)
Platinum	9.43 × 10^6^	(de Podesta 1996)
Saline	1.76	(Horch 2017)
Perineurium	0.0008703	(Pelot et al. 2019; Weerasuriya et al. 1984)
Endoneurium	{0.167, 0.167, 0.571}	(Pelot et al. 2019; Ranck and BeMent 1965)

We simulated thresholds for 1000 model A fibers and 1000 model B fibers randomly positioned in the nerve cross section. We assumed a truncated normal distribution of diameters which we restricted to two standard deviations from the mean (A fibers: 7 to 11 μm, B fibers: 2 to 5 μm); we chose diameters based on published fiber diameters in the rodent cervical vagus nerve (Licursi de Alcântara et al. 2008; Stakenborg et al. 2020), and the conduction velocity of these fibers agrees with in vivo recordings from the vagus nerve in pigs and dogs (Nicolai et al. 2020; Yoo et al. 2013). We shifted each fiber longitudinally along the length of the nerve by a random value drawn from a uniform distribution from − 0.5*INL to 0.5*INL (INL: internodal length, i.e., distance between the nodes of Ranvier for that fiber diameter).

We simulated thresholds for fibers in response to a 300 μs per phase biphasic symmetric rectangular pulse with no delay between phases to match the waveform that was used in vivo. Using a bisection search algorithm (1% tolerance), we solved for threshold current amplitudes required to initiate an action potential in each fiber. We detected action potentials (i.e., V_m_ passing − 30 mV with a rising edge) at 90% of the fiber length, near the end of the fiber distal to the cathode-leading electrode contact.

### Statistics

Statistical analyses were performed using JMP Pro v15.0.1 (SAS Institute Inc., Cary, NC, USA) and Microsoft Excel v16.64 (Microsoft Corporation, Redmond, Washington, USA). Outcomes were analyzed using omnibus 2-way analysis of variance (ANOVA) for constant frequency stimulation (frequency and amplitude), omnibus 3-way ANOVA for burst patterned VNS (inter-burst frequency, mean pulse rate, and amplitude), and single ANOVA for individual factors where appropriate. Tukey post-hoc tests with Bonferroni corrections were used to compare mean responses to responses to 1.0xBCT, 20 Hz, which was used for defining BCT and for EMG normalization. We performed an effect size analysis by calculating the sum of squares (SS) for factors and interactions. We calculated percent variability explained by a given factor by dividing the factor SS by the grand SS (all data points). We performed linear regressions using MATLAB, and coefficient of determination and *p*-values were computed using the fitlm( ) function. All data are reported as means ± standard deviations unless otherwise stated.

## Results

### In vivo experiments

We conducted acute experiments in sevoflurane-anesthetized mice to quantify the effects of VNS on HR and EMG. All analyses of effects of stimulation parameters included data from at least 8 animals.

#### Physiological responses to constant-frequency stimulation

**Fig. 2:**
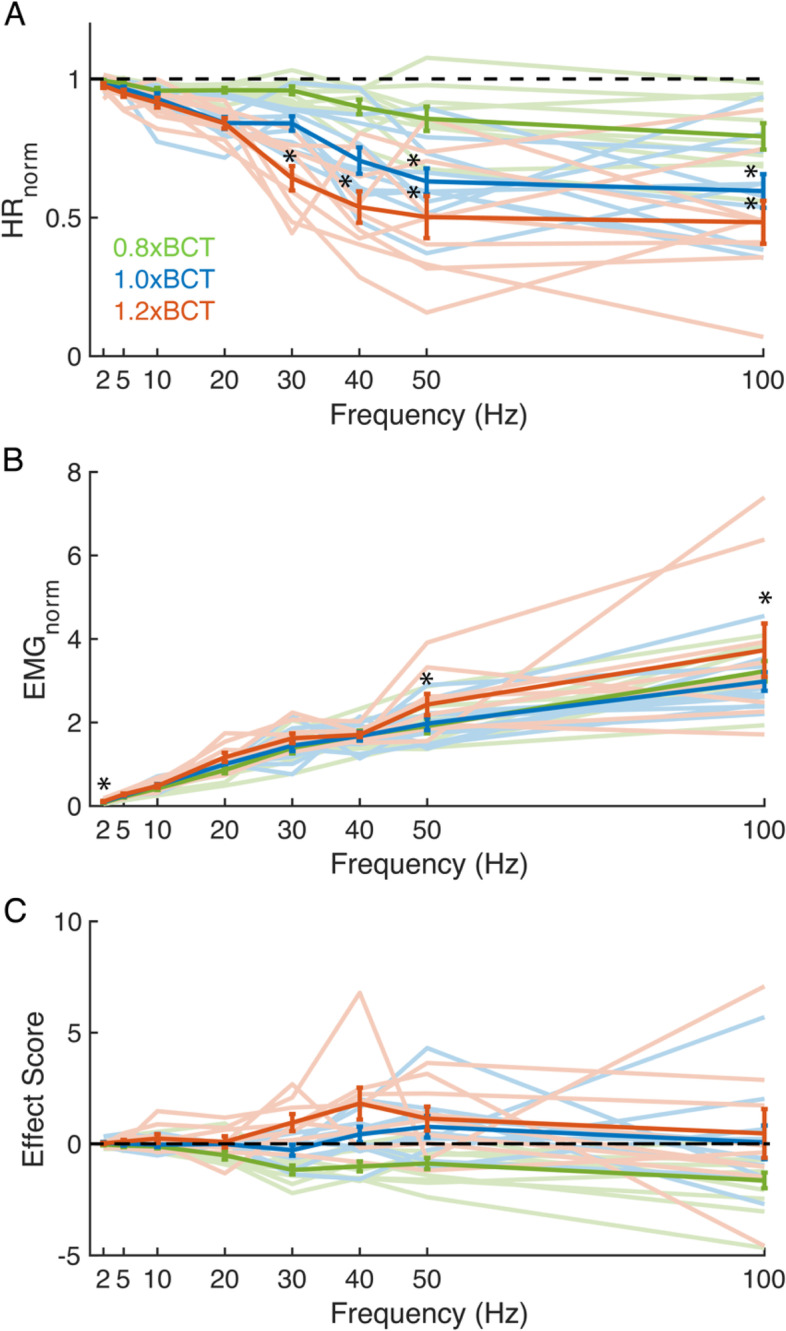
In vivo responses to constant frequency stimulation (intra-burst frequency (Hz) equals mean pulse rate (pulses/s)) of the mouse right cervical vagus at amplitudes of 0.8xBCT (green), 1.0xBCT (blue), and 1.2xBCT (orange). **A** Heart rate normalized to pre-stimulation baseline (HRnorm). **B** EMG normalized to response from stimulation at 1.0xBCT, 20 Hz (EMGnorm). **C** Effect score combining both heart rate and muscle responses normalized to response from stimulation at 1.0xBCT, 20 Hz. Data are presented as mean ± standard error with individual animals as light lines, *n* = 9-10/parameter set. The standard errors at 20 Hz for 1.0xBCT are 0 because those data points were used for normalization. *p < 0.05 in Tukey post-hoc test, comparing to mean responses to stimulation at 1.0xBCT, 20 Hz

We quantified the effects of constant frequency VNS on HR and EMG (Fig. 2). BCT was defined (median: 0.12 mA, range: 0.04 to 0.8 mA) with a constant frequency of 20 Hz to produce a 10-15% reduction in HR (HR_norm_ = 0.84 ± 0.07) (Fig. 2A). HR was reduced more by higher stimulation frequencies and amplitudes, and the effects of frequency on HR_norm_ plateaued between 50 and 100 Hz for all three amplitudes. After omnibus ANOVA test (*F*(41,182) = 14.5, *p* < 0.0001), we found that HR_norm_ was sensitive to stimulation amplitude (*F*(2,221) = 21.0, *p* < 0.0001) and frequency (*F*(7,216) = 25.5, *p* < 00.001), but not the interaction between amplitude and frequency (*F*(23,200) = 0.9, *p* = 0.617). Animal was not a significant factor (*F*(9,214) = 1.5, *p* = 0.141).

We compared HR_norm_ for each parameter set to the response to 1.0xBCT at 20 Hz stimulation. At 1.0xBCT, stimulation at 50 Hz (0.63 ± 0.15, *p* = 0.00926) and 100 Hz (0.60 ± 0.19, *p* < 0.000567) reduced HR_norm_ compared to 20 Hz stimulation, but not 30 Hz or 40 Hz. At 1.2xBCT, stimulation at 30 Hz (0.64 ± 0.13, *p* = 0.0297), 40 Hz (0.53 ± 0.17, *p* < 0.0001), 50 Hz (0.50 ± 0.23, *p* < 0.0001), and 100 Hz (0.48 ± 0.23, *p* < 0.0001) reduced HR_norm_ compared to 1.0xBCT 20 Hz stimulation (Additional file 1: Table 4). No frequency and amplitude combination increased HR_norm_ compared to 1.0xBCT 20 Hz.

EMG_norm_ monotonically increased with stimulation frequency (2 to 100 Hz) for all amplitudes but changed little across amplitudes for a given frequency (Fig. 2B). After omnibus ANOVA test (*F*(41,182) = 22.3, *p* < 0.0001), we found that EMG was sensitive to frequency (*F* = 117.9, *p* < 0.0001), but not stimulation amplitude or the amplitude-frequency interaction (*F*(2,221) = 0.8, *p* = 0.443; *F*(23,200) = 0.08, *p* = 0.999 respectively). We observed a slight increase in average EMG_norm_ for 1.2xBCT compared to 0.8xBCT and 1.0xBCT at 50 and 100 Hz, but this was driven by two outliers. Animal was not a significant factor (*F*(9,214) = 0.6, *p* = 0.806).

We compared EMG_norm_ for each parameter set to the response to 1.0xBCT at 20 Hz stimulation. Post hoc analysis revealed that 2 Hz evoked smaller EMG_norm_ compared to 1.0xBCT 20 Hz for all stimulation amplitudes (0.8xBCT: *p* = 0.0320, 1.0xBCT: *p* = 0.0296, 1.2xBCT: *p* = 0.0476) (Additional file 1: Table 5). Conversely, 50 and 100 Hz evoked higher EMG_norm_ at all amplitudes (50 Hz, 0.8xBCT: *p* = 0.0359, 1.0xBCT: *p* = 0.0103, 1.2xBCT: *p* < 0.0001; 100 Hz, 0.8xBCT: *p* < 0.0001, 1.0xBCT: *p* < 0.0001, 1.2xBCT: *p* < 0.0001). We did not observe different EMG_norm_ at 5 Hz, 10 Hz, 30 Hz, or 40 Hz at any amplitude when compared to 1.0xBCT, 20 Hz. The similarity across amplitudes is consistent with the ANOVA findings that EMG_norm_ was insensitive to amplitude and reports of EMG saturation at BCT in dogs (Yoo et al. 2016) and pigs (Nicolai et al. 2020), indicating EMG saturation over the tested range of stimulation amplitudes (0.8xBCT-1.2xBCT).

The effect score quantified the relationship between VNS-evoked changes in HR and laryngeal muscle activation where positive values were associated with the physiological proxy for therapeutic effects (decreased HR_norm_) and negative values were associated with side effects (increased EMG_norm_) (Fig. 2C). We did not observe obvious monotonic trends across stimulation frequency for the effect score, although frequency effects were clear for HR_norm_ and EMG_norm_ separately. Higher amplitudes produced higher effect scores for frequencies above 20 Hz. After omnibus ANOVA test (*F*(41,182) = 4.7, *p* < 0.0001), we found that the effect score increased with amplitude (*F*(2,221) = 20.2, *p* < 0.0001). Neither frequency nor the amplitude-frequency interaction influenced the effect score (*F*(7,216) = 1.1, *p* = 0.390; *F*(23,200) = 1.1, *p* = 0.403, respectively). No combination of amplitude and frequency produced a different effect score when compared to 1.0xBCT, 20 Hz (0 by definition) in post hoc analysis (Additional file 1: Table 6). We unexpectedly observed an effect of animal (*F*(9,214) = 3.2, *p* = 0.0011), in contrast to HR_norm_ and EMG_norm_ outcomes. Data from a single animal (XX9) exhibited comparatively lower effect scores and its exclusion removed the effect of animal. In summary, HR_norm_ decreased with increased stimulation frequency and amplitude, EMG_norm_ increased with increased frequency, and Effect Score increased with increased amplitude.

#### Physiological responses to temporal patterns of stimulation

**Fig. 3:**
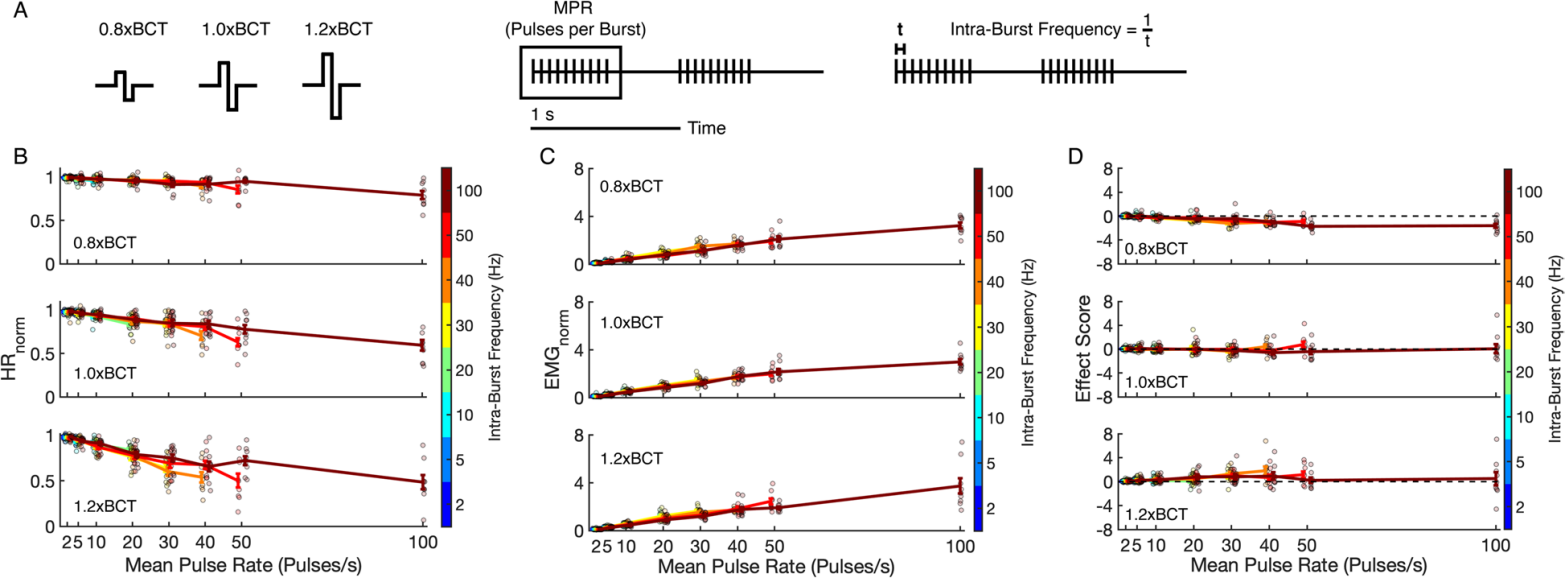
In vivo responses to burst patterns of VNS at amplitudes of 0.8xBCT (top row), 1.0xBCT (middle row), and 1.2xBCT (bottom row) across mean pulse rates (x-axis) and intra-burst frequencies (color). **A** Illustration of stimulation parameters, including amplitude (0.8xBCT, 1.0xBCT, and 1.2xBCT), mean pulse rate (MPR), and intra-burst frequency. **B** Heart rate normalized to pre-stimulation baseline (HR_norm_). **C** EMG normalized to response from stimulation at 1.0xBCT, 20 pulses/s, 20 Hz (EMG_norm_). **D** Effect score combining both heart rate and EMG responses normalized to the response from stimulation at 1.0xBCT, 20 pulses/s, 20 Hz. Data are presented as mean ± standard error with individual data points as points, *n* = 8-10/parameter set

We designed a set of burst patterns to probe the importance of temporal pattern on VNS outcomes. Burst patterns differentially modulated the three outcomes (HR_norm_, EMG_norm_, and effect score). After omnibus ANOVA test (*F*(106,900) = 37.8, *p* < 0.0001), HR_norm_ was sensitive to amplitude, frequency, and MPR (*F*(2,1004) = 81.5, *p* < 0.0001; *F*(7,999) = 4.0, *p* = 0.0002; *F*(7,999) = 80.6, *p* < 0.0001, respectively, Fig. 3A). However, these findings were qualified by interactions between amplitude and MPR (*F*(23,983) = 4.3, *p* < 0.0001), frequency and MPR (*F*(35,971) = 1.8, *p* = 0.0023), but not amplitude and frequency (*F*(23,983) = 0.6, *p* = 0.930).

HR_norm_ decreased with increased amplitude or increased MPR, and higher amplitudes increased the effect of MPR on HR, as observed in response to constant frequency VNS (Fig. 2). Generally, constant frequency stimulation produced greater changes in HR than burst patterns of equal MPR. Reduced changes in HR with burst frequencies compared to constant frequencies of equivalent MPR was most notable at 1.2xBCT, lessened at 1.0xBCT, and abolished at 0.8xBCT. Compared to 1.0xBCT, stimulation with 0.8xBCT did not decrease HR_norm_, stimulation MPR values greater than 40 pulses/s could decrease HR_norm_ at 1.0xBCT, and stimulation MPR values as low as 30 pulses/s could reduce HR_norm_ at 1.2xBCT (*p* < 0.05, Additional file 1: Table 4).

Higher intra-burst frequency decreased the effect of MPR on HR, and this was not an effect exclusively of burst duration and pause duration. For example, patterns with equal burst duration and pause duration (0.5 s burst + 0.5 s pause, i.e., 50% duty cycle) reduced HR_norm_ with increased MPR and amplitude (Additional file 1: Fig. 18). In contrast to findings with constant frequency stimulation, we detected differences across animals (*F*(9,997) = 15.2, *p* < 0.0001).

Burst patterns had effects comparable to constant frequency stimulation on EMG_norm_. After omnibus ANOVA test (*F*(106,896) = 37.1, *p* < 0.0001), EMG_norm_ was dependent on MPR (F7,995) = 295.5, *p* < 0.0001) but not amplitude or intra-burst frequency (*F*(2,1000) = 1.3, *p* = 0.264; *F*(7,995) = 0.7, *p* = 0.667, respectively, Fig. 3B), and none of the interaction terms was significant. In contrast to constant frequency stimulation, inter-individual differences affected EMG_norm_ during burst patterns of stimulation (*F*(9,993) = 7.4, *p* < 0.0001). Given that pattern did not affect the EMG response, we again found patterns with MPR of 2 and 5 produced less muscle activation while greater muscle activation was observed with patterns with MPR of 40, 50, and 100 (along with 1.2xBCT, 30 Hz constant frequency stimulation) (*p* < 0.05, Additional file 1: Table 5).

Effect score was calculated for each trial and normalized to the individual response at 1.0xBCT, 20 Hz (Fig. 3C). With few exceptions, effect scores for burst patterns of stimulation did not differ from constant frequency stimulation at equal MPR. After omnibus ANOVA (*F*(106,896) = 20.6, *p* < 0.0001), the effect score was dependent on amplitude, frequency, and MPR (*F*(2,1000) = 86.3, *p* < 0.0001; *F*(7,995) = 4.6, *p* < 0.0001; *F*(7,995) = 4.8, *p* < 0.0001, respectively), and there was an interaction between amplitude and MPR (*F*(23,979) = 5.7, *p* < 0.0001) but not between amplitude and frequency (*F*(23,979) = 0.7, *p* = 0.866) or frequency and MPR (*F*(35,967) = 1.3, *p* = 0.0851). There was an effect of animal on effect score that was consistent with our other outcomes using burst patterns of stimulation (*F*(9,993) = 51.4, *p* < 0.0001).

Three patterns of stimulation produced effect scores different than 1.0xBCT, 20 Hz constant frequency. Two patterns at 0.8xBCT produced lower effect scores (i.e., emphasized muscle effects over HR effects): 100 Hz constant frequency (effect score = − 1.63, *p* = 0.0152) and 100 Hz, 50 MPR (effect score = − 1.72, *p* = 0.0050). The only pattern to improve effect score was 1.2xBCT, 40 Hz constant frequency stimulation (effect score = 1.82, *p* = 0.0014) in contrast to the lack of significance when the post-hoc test was performed with constant frequency data only (*p* = 0.128). In summary, HR_norm_ decreased with increased stimulation amplitude, increased MPR, and decreased intra-burst frequency; EMG_norm_ increased with increased MPR; Effect Score increased with increased amplitude and decreased intra-burst frequency; and the effect of MPR on Effect Score depended on amplitude.

#### Effect size analysis to determine relative importance of stimulation parameters

**Fig. 4:**
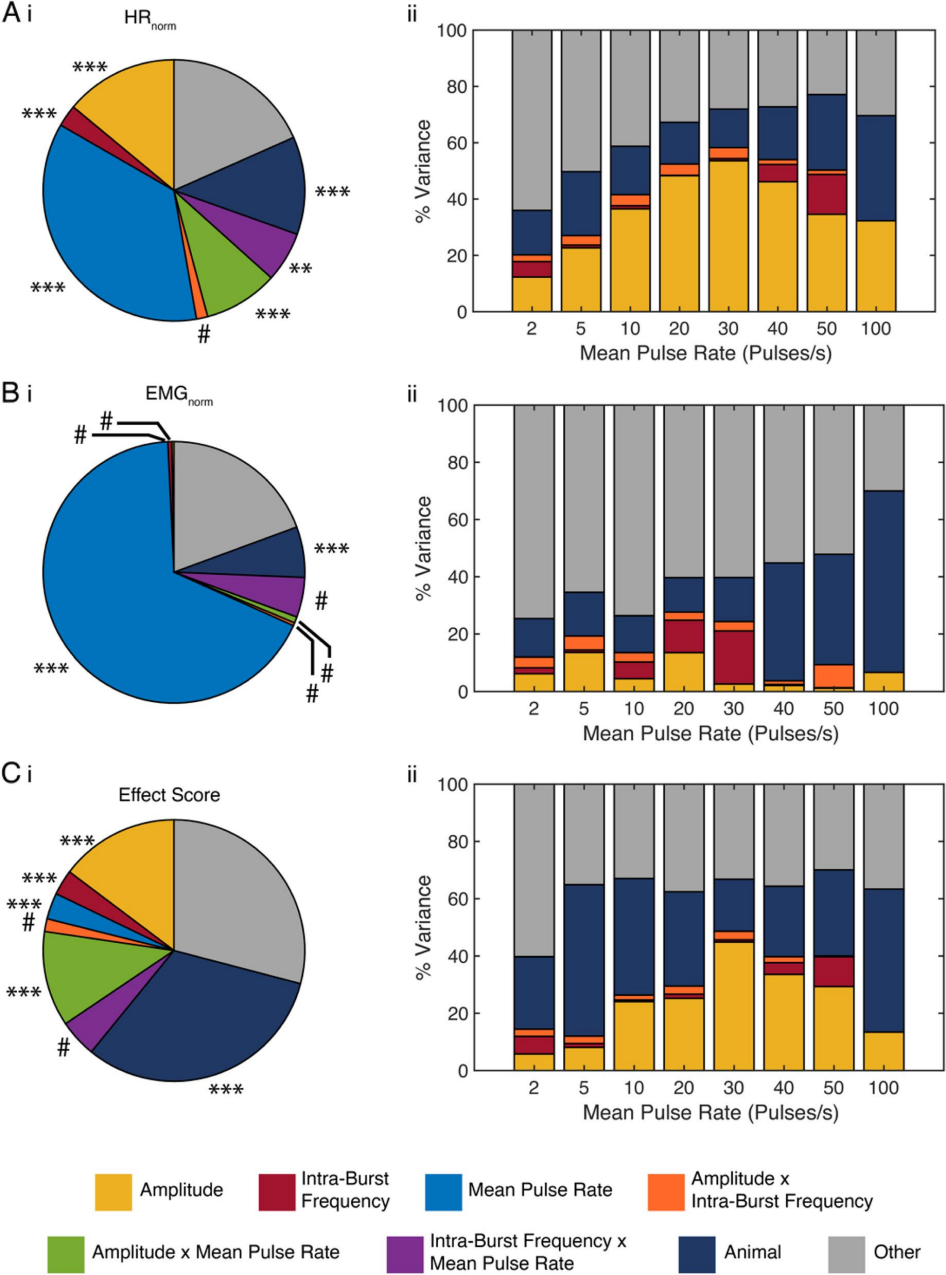
Effect size analysis across VNS parameters (normalized heart rate (HR_norm_), (**A**); normalized EMG (EMG_norm_), (**B**); effect score (**C**), for responses across all burst patterns (left, i) and for responses subdivided by mean pulse rate (MPR; right, ii). Statistical significance was analyzed with a three-way ANOVA, including factors of amplitude (yellow), intra-burst frequency (red), mean pulse rate (blue), interaction between amplitude and frequency (orange), interaction between amplitude and mean pulse rate (green), interaction between frequency and mean pulse rate (purple), animal (dark blue), and unaccounted variance (grey). #*p* > 0.05, **p < 0.01, ***p < 0.001 in multi-way ANOVA

To determine further the contributions of each stimulation parameter to changes in physiological responses, we conducted an effect size analysis (Fig. 4). For HR_norm_, the MPR was the dominant parameter (explained 36% of variance), followed by amplitude (14% of variance) (Fig. 4Ai). We then sub-divided the data by MPR and found the relative contribution of amplitude to be frequency-dependent with a maximum effect at 30 MPR (24% of variance) (Fig. 4Aii).

When the same analysis was applied to EMG_norm_, MPR explained 68% of variance, and across the other stimulation factors, only the interaction term between frequency and MPR contributed more than 1% (6% of variance) (Fig. 4Bi). The effect size of inter-individual variability, i.e., animal, increased with MPR and was largest at 100 MPR (63% of variance) (Fig. 4ii).

MPR explained much less of the variation in effect score when compared to the separate analyses of HR_norm_ and EMG_norm_ (3% of variance compared to 36 and 68%, respectively) (Fig. 4Ci). Rather, inter-animal variability was the dominate factor (32%), suggesting that HR and EMG interacted differently across animals. Frequency and MPR contributed only 3% of variance each. The interaction between amplitude and MPR explained more variance than in the separate analyses of HR_norm_ and EMG_norm_ (12% of variance compared to 9 and 0.8%, respectively). When the data were sub-divided by MPR, the effect size trends followed those observed in HR_norm_, where the effect size of amplitude was dependent on MPR and peaked at 30 MPR (45% of variance) (Fig. 4Cii).

#### Physiological responses to random patterns of stimulation

**Fig. 5:**
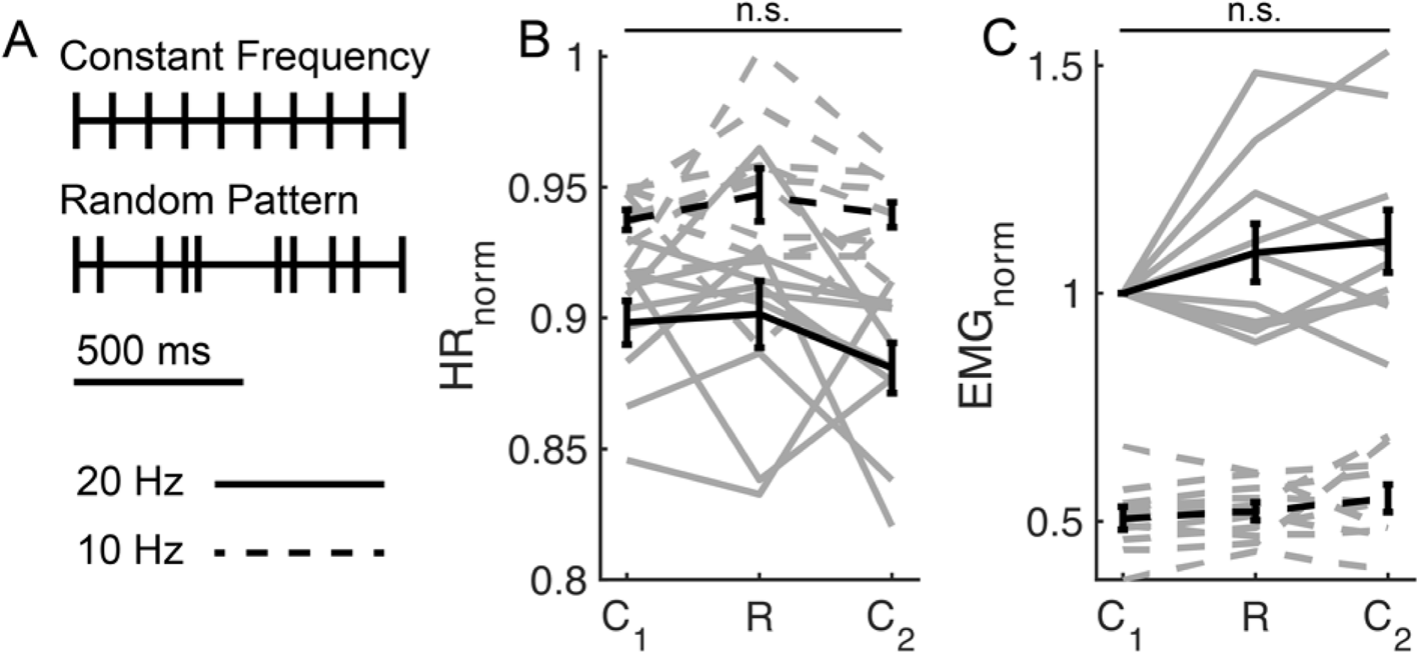
In vivo responses to constant frequency VNS and random patterns of stimulation with an equivalent mean pulse rate of 10 Hz (dashed lines) or 20 Hz (solid lines) at 1.0xBCT. Trials were delivered in a standard order with constant frequency stimulation (C_1_), the trial of random pattern of stimulation (R), and the second trial of constant frequency stimulation (C_2_) for 20 Hz and 10 Hz. Data from individual animals are in gray, and the population response is in black (mean ± standard error; *n* = 10). **A** Example constant frequency and random pattern pulse trains for MPR = 10 pulses/s. **B** Heart rate normalized to pre-stimulation baseline (HR_norm_). **C** EMG normalized to the response during C_1_, 20 Hz stimulation (EMG_norm_). n.s., not significant, *n* = 10

We conducted VNS trials with random patterns of stimulation paired with constant frequency trials that had the same MPR (Fig. 5A). The HR_norm_ and EMG_norm_ responses did not change between the first constant frequency trial (C_1_), the random pattern trial (R), or the second constant frequency trial (C_2_) for MPRs of 10 Hz (*F*(2,27) = 0.76, *p* = 0.477; *F*(2,27) = 1.23, *p* = 0.309, respectively) or 20 Hz (*F*(2,27) = 0.55, *p* = 0.586; *F*(2,27) = 1.13, *p* = 0.339, respectively) (Fig. 5B, C).

To determine if HR_norm_ or EMG_norm_ responses were influenced by pattern frequency characteristics other than MPR, we performed linear regressions between each response and the mean inter-pulse frequency (<IPF>) and the geometric mean IPF (<IPF > _Geom_). Neither outcome correlated with frequency characteristics of patterns (Additional file 1: Fig. 19). While burst patterns altered HR response, these results indicate that random patterns did not affect HR_norm_ nor EMG_norm_ compared to constant frequency of equivalent MPR. In summary, the effects of random patterns of stimulation did not differ from constant frequency stimulation and frequency characteristics of the random patterns (mean IPF, geometric mean IPF) did not influence outcomes.

#### Distal vagotomy abolished VNS-evoked physiological responses

**Fig. 6:**
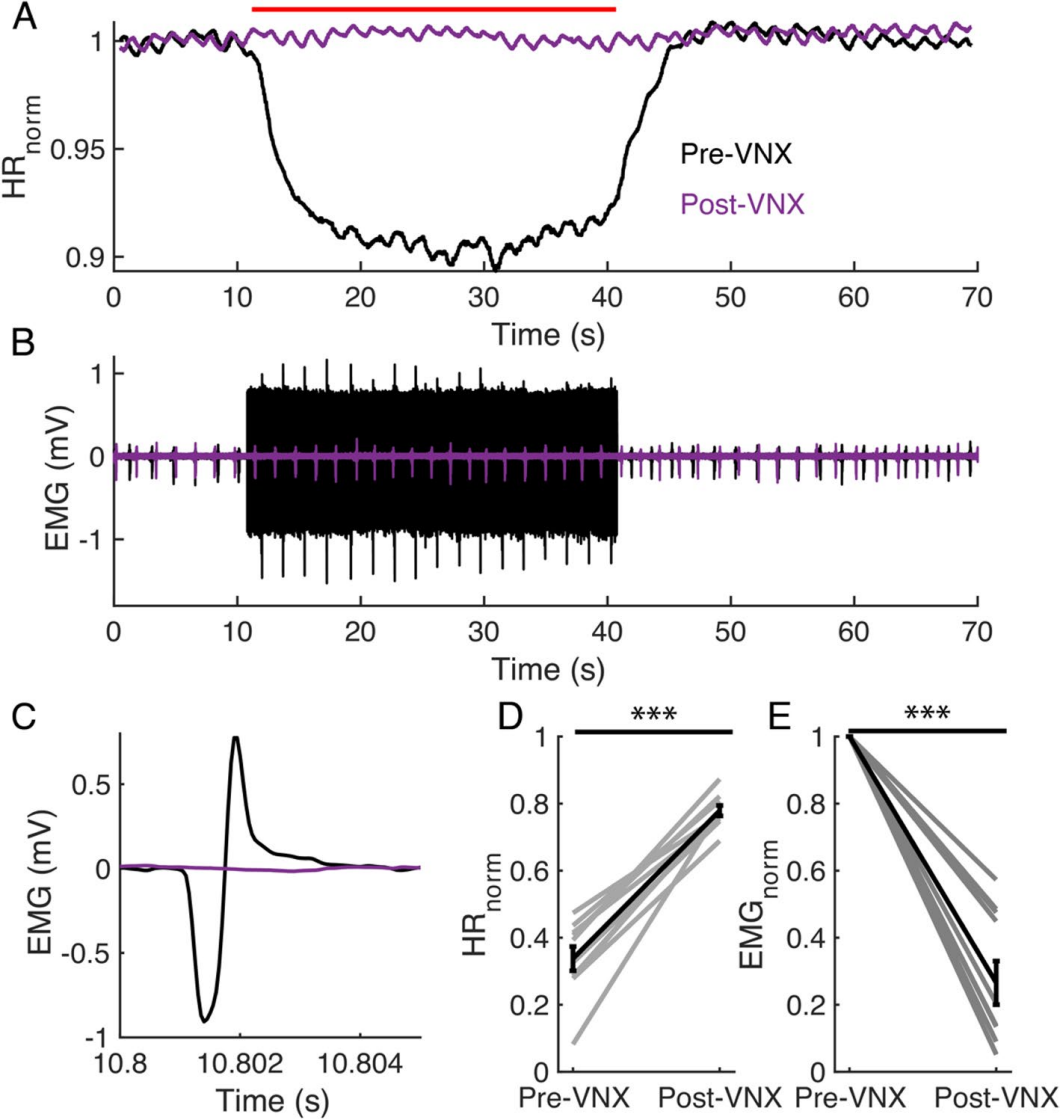
Vagotomy (VNX) distal to the stimulation cuff abolished VNS-evoked responses. **A**-**B** Example heart rate (HR) and EMG responses. Stimulation was applied at 20 Hz at bradycardia threshold (BCT) determined pre-VNX from t = 10 s to t = 40 s (red bar). **A** HR_norm_ pre-VNX (black) and post-VNX (purple). **B** EMG_norm_ pre-VNX (black) and post-VNX (purple). **C** EMG response to a single stimulation pulse pre- and post-VNX. **D** HR_norm_ pre- and post-VNX. **E** EMG_norm_ pre- and post-VNX. *** *p* < 0.0001, *n* = 10

To determine whether VNS-evoked effects were mediated by efferent fibers, we performed an end-of-study vagotomy. We compared HR and EMG during trials of 1.0xBCT, 20 Hz constant frequency stimulation before and after transection of the cervical vagus nerve distal to the stimulation cuff (Fig. 6A-B). Vagotomy abolished HR (*p* < 0.0001, Fig. 6D) and EMG responses to VNS (*p* < 0.0001, Fig. 6E). No evoked EMG waveforms were observed post-VNX (Fig. 6C), and non-zero values of post-transection EMG_norm_ (Fig. 6E) arose from a substantial noise floor for some animals. Therefore, physiological responses were mediated by efferent vagal fiber activation.

### Computational models

#### Parameterization of HR model and response to constant frequency stimulation

**Fig. 7:**
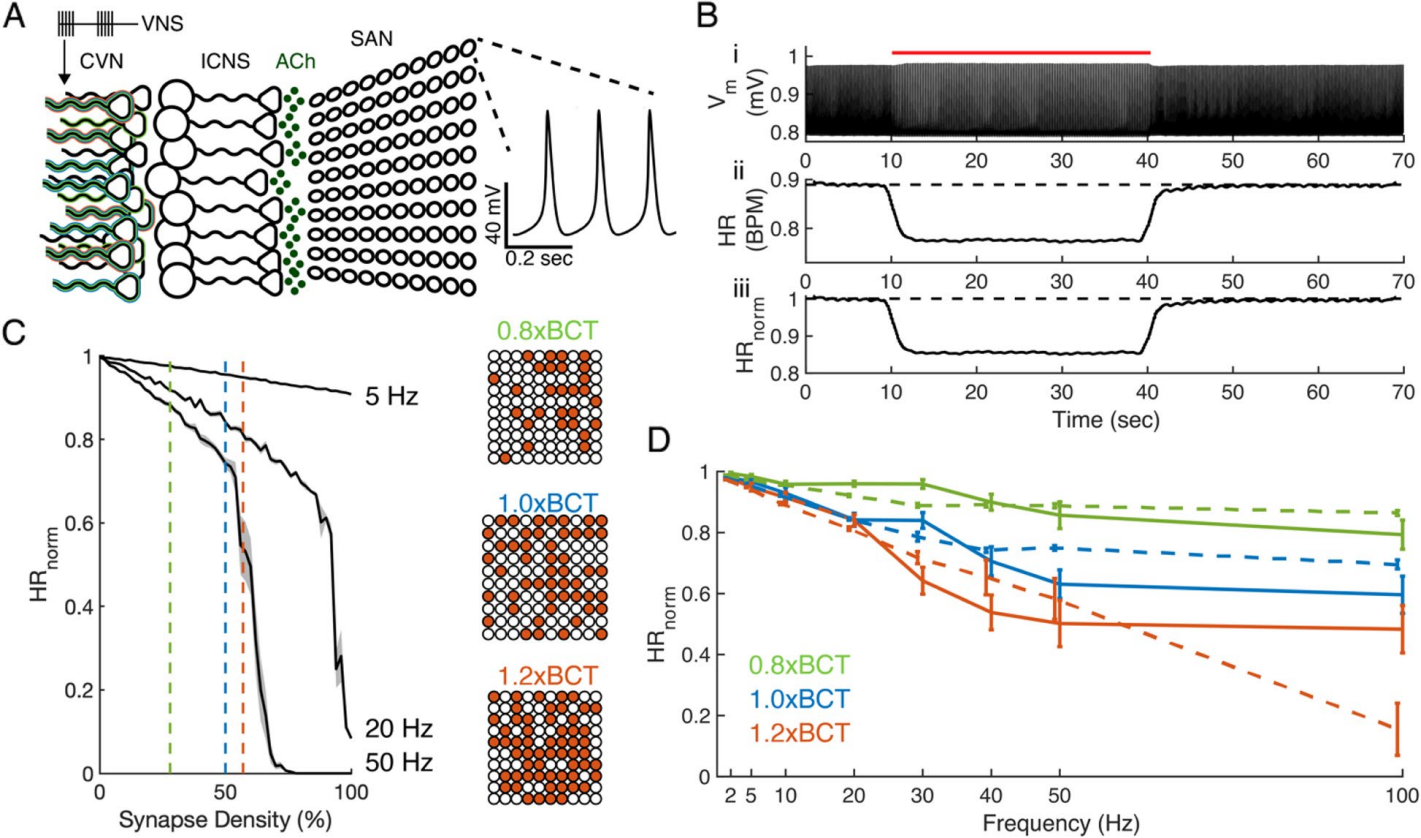
Implementation and validation of a model of VNS effects on HR. **A** Schema of computational model. VNS pattern served as input from the cervical vagus nerve (CVN) to post-ganglionic cells in the intrinsic cardiac nervous system (ICNS). ICNS included mechanisms for frequency-dependent VN-to-ICNS synaptic failure and ICNS intrinsic firing. ACh release from ICNS cells to sinoatrial node (SAN) cells was modeled using a three-compartment model; post-synaptic mechanisms for ACh-dependent modulation of ion channels and internal calcium storage were incorporated in our 10-by-10 network of SAN cells. The network of SAN cells consisted of spontaneously firing cells. The mean firing rate of the SAN cells on the periphery of the 10-by-10 network was interpreted as HR. **B** Model responses where SAN cell firing (i) was used as a proxy for HR (ii). To compare with in vivo data, HR during stimulation (t = 15 s to 40 s) was normalized to pre-stimulation baseline (t = 0 to 10 s) (iii). The first 5 s of stimulation were excluded from analysis due to the transient response in HR. **C** Stimulation amplitude was simulated by adjusting the number of SAN cells receiving VNS-evoked ACh release from the cells of the ICSN (Synapse Density) during constant frequency stimulation (5, 20, and 50 Hz) for mean (black line) and SE (grey shaded area). Higher stimulation amplitudes were implemented as higher synaptic densities. **D** Model outcomes (dashed lines) compared to in vivo data (solid lines). Data are presented as mean ± standard error, *n* = 9-10 per parameter set for in vivo data, *n* = 10 runs per parameter set for model data

We implemented a computational model of CVN-to-ICNS connections, post-ganglionic release of ACh, and ACh-mediated changes to SAN cell firing rate (Fig. 7A). Our implementation of a single mouse-specific SAN cell was validated with published data and publicly available code (Additional file 1: Fig. 15). Trial structure and calculation of HR_norm_ in the model mirrored the in vivo measurements (Fig. 7B). We simulated the response to 5, 20, and 50 Hz constant frequency stimulation and found a monotonic decrease in HR_norm_ with increasing active ACh synapse density (proxy for stimulation amplitude) (Fig. 7C). Higher frequencies produced steeper decreases in HR_norm_ and asystole at ACh synapse densities above 70% at 50 Hz. We identified a synaptic density associated with each stimulation amplitude (0.8xBCT, 1.0xBCT, and 1.2xBCT) using a bisection search method that minimized the error between measured HR_norm_ and modeled HR_norm_ (Table 1). The interaction between amplitude and frequency in modeled outcomes matched trends of measured HR in our in vivo data (Fig. 2A) and was also consistent with prior in vivo studies in dog (Yoo et al. 2016). Model outcomes were within one standard deviation (SD) of in vivo measurements for 92% of cases: 6 of 8 constant frequencies for 0.8xBCT, 8 of 8 constant frequencies for 1.0xBCT, and 8 of 8 constant frequencies for 1.2xBCT (Fig. 7D).

#### Parameterization of muscle activation model and response to constant frequency stimulation

**Fig. 8:**
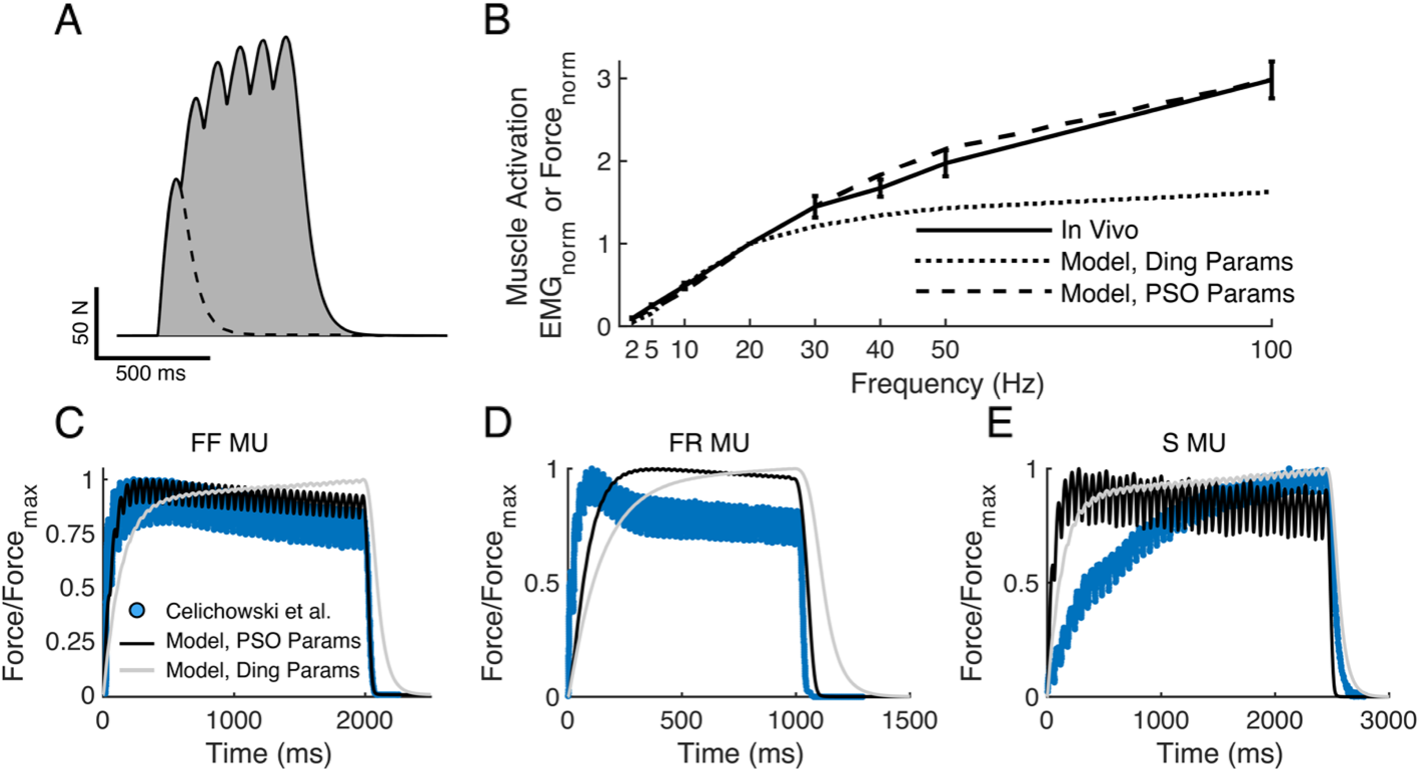
Computational model of VNS-evoked muscle contractions. **A** Modeled force from VNS pulse (dashed line) and pulses (solid line). Model output was quantified by the force-time integral (gray area). Simulation parameters used are from (Ding et al. 2003). **B** Responses to constant frequency VNS in vivo (solid line), model using (Ding et al. 2003) parameters (dotted line), and model using PSO-identified parameters (dashed line). **C**-**E** Model of force production using Ding 2003 parameters (grey) and PSO-identified parameters (black) overlaid on force measurements from different types of skeletal muscle motor units (MU) in the rat medial gastrocnemius (Celichowski et al. 2014). Force was normalized by maximum force. **C** Modeled and in vivo data from fast fatigable MU (FF MU) response to 20 Hz stimulation. **D** Model and data from fast fatigue-resistant motor unit (FR MU) response to 40 Hz stimulation. **E** Model and data from slow MU (S MU) response to 16.6 Hz stimulation

Model parameterization was necessary because model outcomes did not agree with in vivo outcomes when we used published parameter values (identified from studies of human quadriceps muscle, Table 2 and Fig. 8B). We parameterized our muscle activation model using our in vivo EMG responses to 1.0xBCT because stimulation amplitude accounted for only a small proportion of the in vivo data variance of EMG (Fig. 4Bi). We optimized the model parameters against the mean response across animals for 18 randomly selected temporal stimulation patterns, and the PSO-identified model parameter values that generated the lowest model error (Table 2) were used for subsequent simulations.

Model responses were within one SD of in vivo measurements for all constant frequencies tested (Fig. 8B). Further, the simulation-evoked force (Fig. 8C-E, black) matched most closely measurements of force production from fast, fatigable (FF) motor units (Fig. 8C), which are the most prevalent in mouse laryngeal muscles (Hoh 2005), but did not match well with fast, fatigue-resistant (FR) (Fig. 8D) or slow (S) (Fig. 8E) motor unit force production. Conversely, the human quadriceps femoris muscles have a much higher proportion of S motor units and lower proportion of FF motor units than mouse laryngeal muscles (Hoh 2005; Staron et al. 2000). Indeed, model responses using parameters associated with quadriceps femoris muscles (Fig. 8C-E, grey) agreed less with FF motor units (Fig. 8C) and more with S motor units (Fig. 8E).

#### Models replicated physiological responses to broad range of VNS parameters

**Fig. 9:**
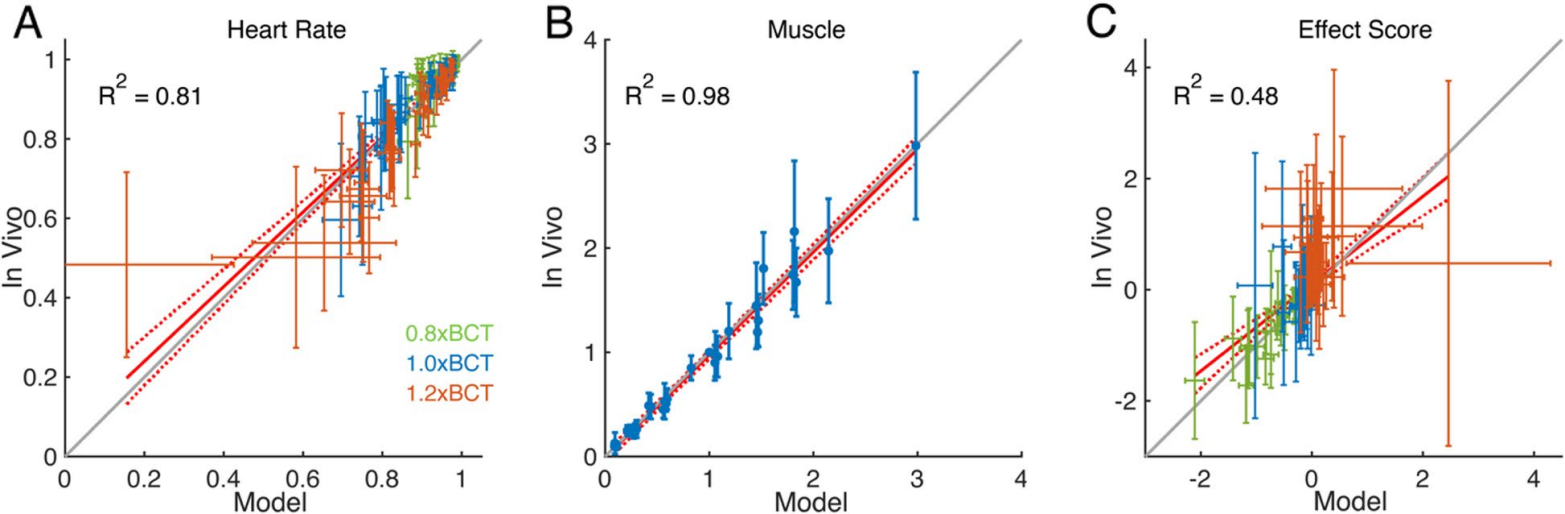
Comparisons of simulated and in vivo data. **A** Comparison of modeled and in vivo HR_norm_ at stimulation amplitudes of 0.8xBCT (green), 1.0xBCT (blue), and 1.2xBCT (orange). **B** Comparison of modeled and in vivo EMG_norm_ and Force_norm_ at only 1.0xBCT. **C** Model effect score calculations used HR_norm_ at the color-coded stimulation amplitudes, but Force_norm_ at only 1.0xBCT. Data are presented as mean ± SD (vertical bars for in vivo SD and horizontal bars for model SD), *n* = 8-10 per parameter set for in vivo data, *n* = 10 runs per parameter set for HR model and *n* = 1 run per muscle model. Coefficient of determination (*R*^2^) of linear fit (solid red line in each panel; 95% confidence interval in dashed red lines in each panel). Gray 1:1 lines represent perfect agreement (in vivo = model)

We quantified model responses to all 108 parameter combinations tested in vivo, across stimulation amplitudes, mean pulse rates, and intra-burst frequencies (Fig. 9). Simulated HR_norm_ was within one SD of the in vivo data for 89% of cases (Fig. 9A and Additional file 1: Table 7), and the largest model errors were HR_norm_ underestimation at 0.8xBCT and 1.0xBCT and HR overestimation at 1.2xBCT. Modeled HR_norm_ followed in vivo trends: higher MPR caused a greater decrease in HR_norm_, lower intra-burst frequency caused a larger decrease in HR_norm_ for a given MPR, and the effect of intra-burst frequency increased with amplitude (Additional file 1: Fig. 20A).

Similarly, modeled muscle activation fell within one SD of the in vivo EMG data in 92% of cases (Fig. 9B and Additional file 1: Table 8) and followed the monotonic relationship between muscle activation (Force_norm_) and MPR observed in vivo (EMG_norm_) (Additional file 1: Fig. 20B). Model error was dependent on intra-burst frequency, with slight overestimation at the lowest and highest intra-burst frequencies and underestimation at intermediate intra-burst frequencies. Across MPRs, the model slightly overestimated muscle activation for intra-burst frequencies between 2 and 10 Hz, underestimated muscle activation between 30 and 50 Hz, and overestimated muscle activation at 100 Hz.

We combined the outcomes from the HR and muscle activation to calculate the effect score as we did for the in vivo data (Fig. 9C and Additional file 1: Table 9). As the muscle model did not incorporate stimulation amplitude, we modeled stimulation amplitude in the HR model only and used the muscle model outcomes from 1.0xBCT to calculate the effect score at all three amplitudes (Additional file 1: Fig. 20C). The modeled effect score increased with increased stimulation amplitude, reproducing in vivo trends, and effect scores were within one SD of the in vivo data in 98% of cases (Fig. 9C). The sign of the effect score is a key metric for interpreting whether stimulation parameters increase cardiac response over muscle response (positive) or vice versa (negative), and the sign of the mean model effect scores matched in vivo values in 77% of cases.

#### Model performance suffers when ICNS mechanism was removed

Although computational models are useful for designing optimal temporal patterns of stimulation (Brocker et al. 2017; Cassar et al. 2017), the small effect size of intra-burst frequency and the lack of difference between response to random versus constant frequency patterns indicate that HR, muscle activation, and the effect score were insensitive to the temporal pattern of VNS. Therefore, we used the model to determine the contributions of VN-ICNS synaptic filtering to the HR response to VNS.

**Fig. 10:**
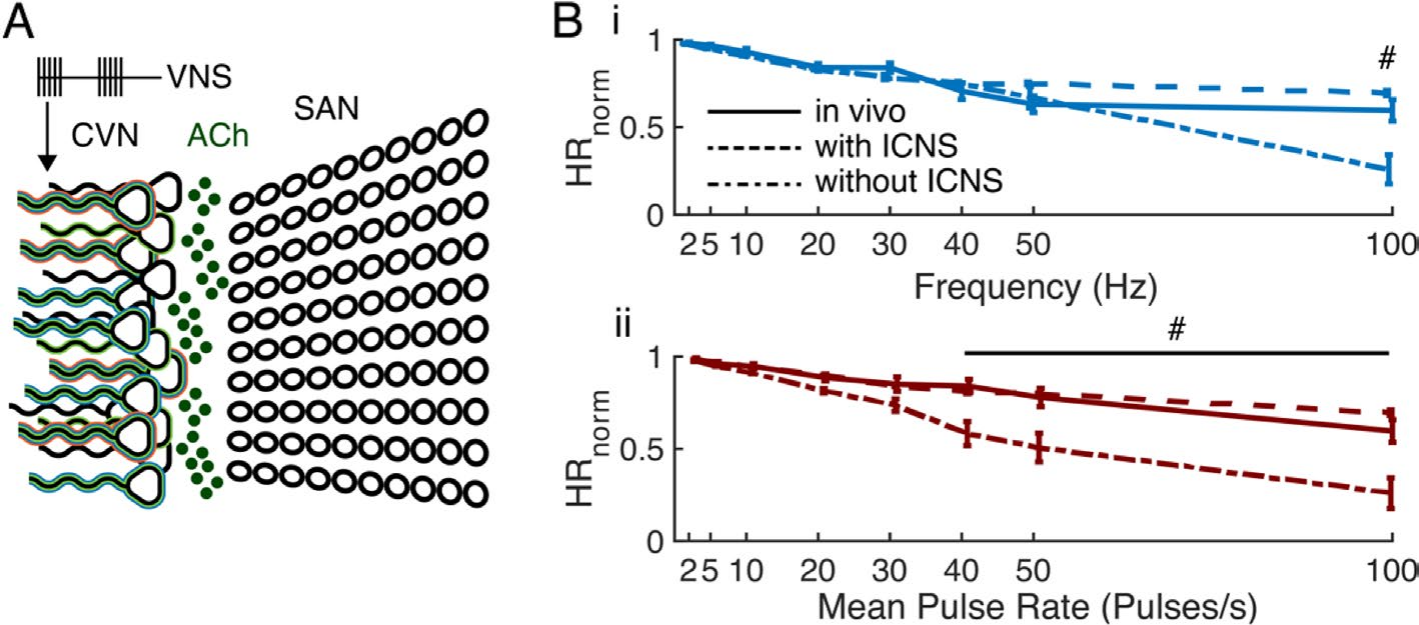
Model perturbations cause the model response to deviate from in vivo data. **A** Diagram of the computational model of the connection of the cervical vagus nerve (CVN) to the sinoatrial node (SAN) of the heart without the intrinsic cardiac nervous system (ICNS) (Fig. 7A). Stimulation pulses (VNS) cause release of acetylcholine (ACh) to the SAN cells. **B** Normalized heart rate (HR_norm_) response to VNS for in vivo data at bradycardia threshold (solid line), original model with ICNS (dashed line), and model with no ICNS (dot-dashed line) for constant frequency stimulation (i) and burst patterns with intra-burst frequency of 100 Hz (ii). Data presented as mean ± standard error, ^#^difference between modeled HR_norm_ and measured HR_norm_ exceeded one standard deviation of in vivo data, *n* = 8-10 per parameter set for in vivo data, *n* = 10 runs per parameter set for computational models

We removed the frequency-dependent ICNS filtering, and thus each VNS pulse caused ACh release to the SAN (Fig. 10A). We then simulated HR in response to constant-frequency VNS at 1.0xBCT. The modified model matched in vivo measurements up to 50 Hz but greatly overestimated the bradycardia effect at 100 Hz (Fig. 10Bi). Further, the modified model overestimated bradycardia for MPR values > 30 pulses/s (Fig. 10Bii), indicating that the frequency-dependent filtering at VN-ICNS synapses was critical to replicate changes in HR in response to VNS pulses delivered at intra-burst frequencies > 50 Hz.

### Extended amplitude range produced overlap of dynamic range of HR and EMG

**Fig. 11:**
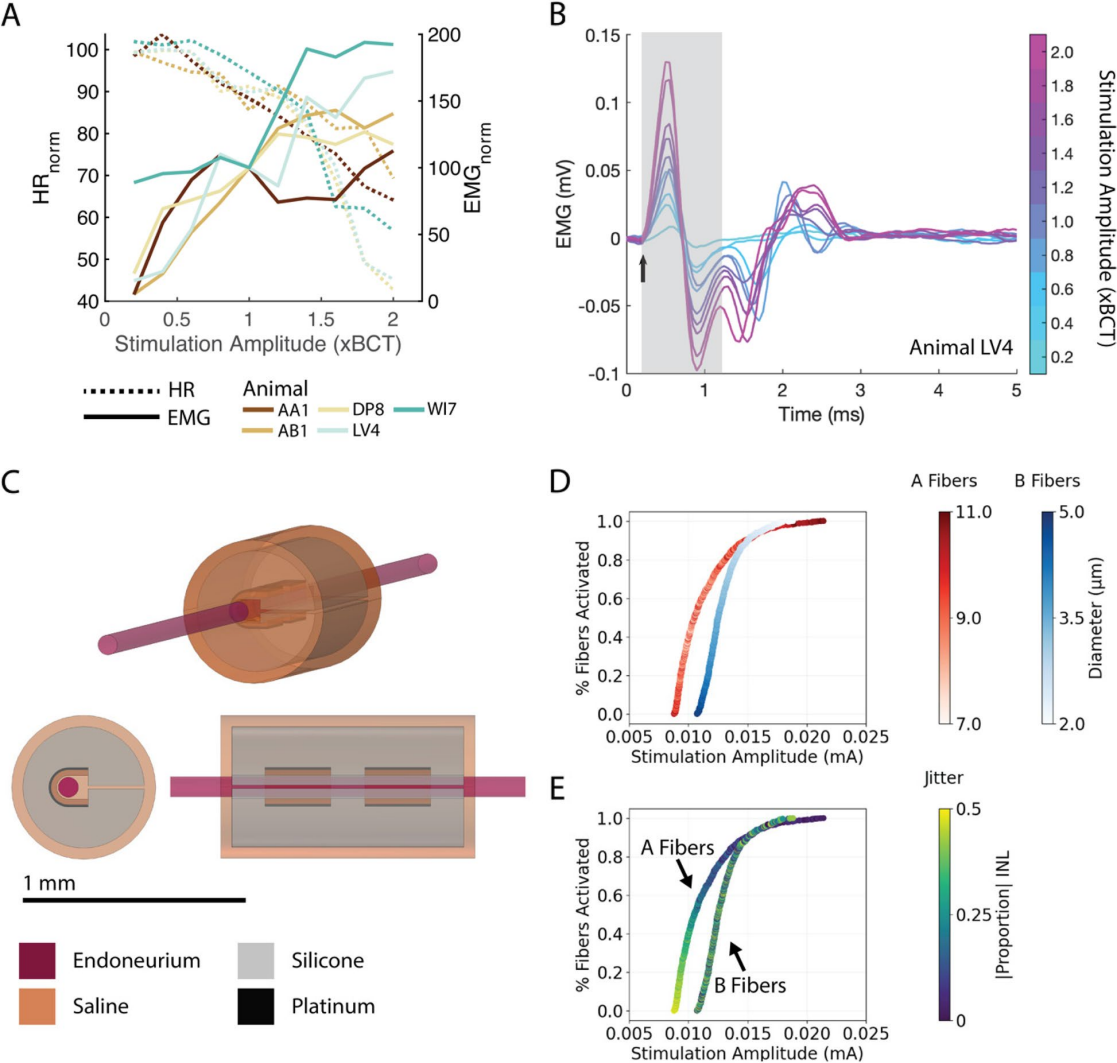
Stimulation amplitude affected heart rate (HR) and laryngeal muscle activation (EMG) in vivo and in computational models. **A** Normalized heart rate (HR; dashed lines) decreased with increased amplitude for all animals. Onset of changes in heart rate occurred at ~ 0.6xBCT (BCT = bradycardia threshold). Normalized EMG responses (solid lines) across stimulation amplitudes varied between animals. Onset of EMG response occurred at ~ 0.4xBCT to 0.6xBCT, and the normalized EMG increased with stimulation amplitude (*n* = 5 animals). Color denotes animal. **B** Illustrative EMG signals from one animal (LV4) in response to a single pulse delivered at different stimulation amplitudes (color). Stimulation onset and artifact are represented as an arrow and grey box, respectively. Increased stimulation amplitude resulted in shorter EMG latency, change in EMG waveform, and merging of EMG and stimulation artifact. **C** Finite element model of the mouse vagus nerve and bipolar cuff electrode. The perineurium sheath around the fascicle and the surrounding muscle are not shown. **D** Recruitment curves for model A and B fibers colored to indicate fiber diameter. **E** Recruitment curves for model A and B fibers colored to indicate fiber jitter (i.e., the relative position of the center node of Ranvier along the length of the nerve)

In the in vivo recordings described above, the EMG signal was saturated across all VNS amplitudes (0.8xBCT to 1.2xBCT). Therefore, we investigated the effects of a broader range of stimulation amplitudes, from 0.2xBCT to 2.0xBCT (*n* = 5, Fig. 11). Bradycardia onset occurred at ~ 0.6xBCT and HR_norm_ monotonically decreased with increased stimulation amplitude (Fig. 11A). EMG_norm_ showed less clear trends across animals: onset occurred at 0.4xBCT (except for one animal with measurable EMG at 0.2xBCT), the signal increased sharply until 0.8xBCT, and it then either plateaued or continued to increase with increased amplitude. The EMG waveform changed with stimulation amplitude (Fig. 11B), indicating that responses included activation of additional motor units.

We paired a finite element model of the mouse vagus nerve and cuff electrode (Fig. 11C) with biophysical models of myelinated fibers to investigate overlap in the dynamic ranges of physiological outcomes. We found strong agreement between model A fiber thresholds and in vivo EMG_norm_ thresholds for 3 of 5 animals (Additional file 1: Fig. 21A) and between B fiber thresholds and in vivo bradycardia onset (Additional file 1: Fig. 21B). However, the model underestimated saturation of B fibers since the HR continued to decrease in vivo even at the highest amplitudes tested.

Recruitment order of the modeled fibers was driven by longitudinal fiber alignment for A fibers (Fig. 11E) and by fiber diameter for B fibers (Fig. 11D). Therefore, although the B fibers were smaller in diameter and smaller fibers generally have higher activation thresholds (Woodbury and Woodbury 1990), the modeled A and B fiber thresholds had overlapping ranges (Fig. 11D-E), consistent with the in vivo recordings (Fig. 11A). Thus, the longitudinal alignment of the fibers’ nodes of Ranvier with respect to the electrode contacts provides a mechanism of recruitment order that is not solely driven by fiber diameter, causing overlap of dynamic ranges of neural responses between populations of nerve fibers in the mouse CVN that are distinct in function and relative excitability.

## Discussion

We quantified the effects of VNS parameters on physiological responses in vivo and in a computational model. We measured VNS-evoked changes in HR and laryngeal EMG in anesthetized mice; MPR had the greatest effect, and bradycardia and EMG increased with increased MPR. Intra-burst frequency affected HR, but the effect size was modest (2.7% of variance explained) and contributed more to variance with increased MPR at values above those used clinically (40 and 50 pulses/s). EMG was influenced by the number of pulses applied per second (frequency with constant frequency and MPR with burst patterns) but insensitive to intra-burst frequency and amplitude over the tested range. Taken together, these findings indicate that our stimulation amplitudes produced saturation (or near saturation) of A fibers and were within in the dynamic range of B fiber activation.

We defined the effect score to quantify the difference between HR and EMG responses to VNS parameters. MPR explained the largest portion of variance among all factors for HR and EMG, but MPR had little impact on effect score. This discrepancy between individual measured outcomes and the combined effect score demonstrates that MPR drives the physiological responses to stimulation but cannot be adjusted in isolation to alter independently HR or muscle activation. Rather, amplitude adjustments were also required to alter effect score, likely due to the high effect size of amplitude on heart rate and low effect size on EMG. We identified 1.2xBCT at 40 Hz to be the single parameter combination to influence HR over EMG as defined by the effect score (effect score = 1.82), compared to 1.0xBCT at 20 Hz (Cohen’s d = 2.38). Our effect score metric for VNS optimization differs from comparable studies. For example, a study in dogs identified burst patterns of stimulation that produced bradycardia with significantly lower laryngeal EMG (Yoo et al. 2016). Alternatively, a “selectivity index” was defined in rats where B fiber selectivity was achieved using high stimulation intensities and longer pulse widths (Chang et al. 2022). Choice of optimization paradigm may be defined by the goals and limitations of the application; for example, parameter adjustment may aim to decrease laryngeal EMG from a defined baseline (e.g., 20-30 Hz), minimize bradycardia, or maximize bradycardia.

We observed a plateau in changes in HR and reduced rate of increase in EMG at higher MPR. Similar studies in rats have noted plateaued or decreased bradycardia effects at frequencies above 50 Hz (Chang et al. 2022; Hotta et al. 2009). While conduction failure of peripheral nerve axons is possible (Zhu et al. 2009), HR and EMG non-linearity at high MPR could be explained through ganglionic fidelity and fatigue, respectively. Frequency-dependent synaptic failure between CVN fibers and ICNS cells occurs in rats, and higher stimulation frequencies resulted in lower fidelity (Rimmer and Harper 2006). Indeed, the model substantially overestimated the reductions in heart rate at higher frequencies and MPRs when we removed synaptic failure, indicating that the ICNS network is a key factor in frequency-dependent VNS-induced changes in HR. The one-to-one ICNS relay network was informed by studies in rats (McAllen et al. 2011; Rimmer and Harper 2006), but the frequency effects may not be conserved across species. For example, ICNS cells in pigs do not receive direct synaptic connections to the soma, but have disperse dendritic arbors (Hanna et al. 2021), requiring integration of multiple preganglionic inputs which may influence ganglionic fidelity.

A similar non-linearity was observed in the frequency response of EMG recordings. This non-linearity may be due to the force-frequency relationship of muscles whereby force output is saturated at increased stimulation frequencies. Our quantification of in vivo muscle activation summed all EMG signals during the trial. If each evoked EMG signal was identical, we would expect a linear relationship between stimulation frequency and EMG. However, the evoked EMG response fell below this linear line at higher frequencies (40-100 Hz) due to EMG waveform changes during stimulation (Additional file 1: Fig. 22). During high frequency trials, evoked EMGs declined in amplitude, indicative of muscle fatigue (Ibitoye et al. 2014). Clinically relevant frequencies (10-30 Hz constant frequency stimulation) would not likely produce the same fatigue effects, but muscle fatigue produced by high frequency stimulation should be considered with new stimulation paradigms such as micro-burst (250-300 Hz) (de la Garza et al. 2019; Martlé et al. 2014).

We used 1 s epochs for burst patterns and random patterns of stimulation based on previous literature (Yoo et al. 2016) and did not explore different epoch durations. Intrinsic vagal and ICNS activity produces fluctuations of HR linked to respiration (~ 60 breaths per minute in anesthetized mice) (McAllen et al. 2011; Simms et al. 2007), i.e., the respiratory sinus arrhythmia. While we applied VNS agnostic to intrinsic vagal activity, future efforts should investigate the effects of VNS timing with respiration, an approach used in other peripheral nerve stimulation applications (Heiser et al. 2019). Timed application of VNS during periods of reduced intrinsic vagal activity (inhalation) could reduce average HR and lower HR variability while stimulation during periods of increased intrinsic vagal activity (exhalation) could lower the minimum instantaneous HR and increase HR variability.

Experiments were conducted under sevoflurane, which produces a notable decrease in resting HR (Cesarovic et al. 2010) but does not influence muscle activity during isometric contraction (Ginz et al. 2004). In rats, the stimulation amplitude to produce a 5-10% reduction in HR was lower when the animal was awake than under isoflurane anesthesia (Ahmed et al. 2021), and the relationship with MPR, intra-burst frequency, and amplitude may be different in awake animals. General anesthesia dampens sympathetic activity (Wood 1994) and modulates reflexive activity from excitation of vagal afferents (Ahmed et al. 2021; Ardell et al. 2017).

Anatomical differences in vagal innervation of the heart informs selection of which side VNS is delivered. Stimulation of the right CVN produces greater changes in HR compared to stimulation of the left CVN in rats (Stauss 2017) and humans (Premchand et al. 2014). We applied stimulation to the right CVN, which is used in clinical trials for treatment of HF (De Ferrari and Schwartz 2011), but is avoided in treatment of epilepsy where cardiac effects of VNS are considered unwanted side effects (Yuan and Silberstein 2016). However, similar bradycardia effects were observed in rats (Hotta et al. 2009) and pigs (Yamakawa et al. 2014), casting doubt on strict guidelines for lead placement to induce or avoid cardiac effects from stimulation.

We confirmed that changes in HR and EMG were mediated by vagal efferent fibers by distal transection of the vagus nerve. Stimulation of the proximal trunk did not induce tachycardia nor did stimulation of the intact nerve with low amplitudes. Conversely, VNS studies in dogs observed tachycardia during stimulation which was purported to arise through activation of sympathetic fibers encased with the CVN in the carotid sheath (Yoo et al. 2016) or reflexive activation of sympathetic mechanisms mediated by vagal afferents (Ardell et al. 2015). Sympathetic efferents within the cervical vagus nerve have been identified in cats (Agostoni et al. 1957), dogs (Onkka et al. 2013), and humans (Seki et al. 2014) but not yet in mice.

We implemented computational models of VNS-induced changes in HR and laryngeal muscle activation that reproduced in vivo responses to temporal patterns. We included amplitude-dependent changes in HR response by altering the number of SAN cells receiving ACh release in response to stimulation pulses; the model slightly overestimated the response (lower simulated HR_norm_ compared to in vivo data) for 0.8xBCT and 1.0xBCT while the model underestimated the response at 1.2xBCT. This could be a result of the parameterization method for determining ACh synapse density. The bisection search method was constrained to data from constant frequency stimulation trials including 100 Hz. At these higher frequencies, the model could simulate asystole at sufficient ACh synapse density (50 Hz synapse density curve, Fig. 7C) and bias the search method. VNS can produce asystole, but likely at greater stimulation intensities than predicted by the model. Improved model performance at high stimulation frequencies may require integration of sympathetic reflexes, as low HR engages compensatory sympathetic mechanisms to increase HR.

While we stimulated the source of parasympathetic efferent cardiac fibers, we did not investigate the contribution of sympathetic modulation of HR. Primary sympathetic innervation to the ICNS and cardiac myocytes is supplied by the stellate ganglia (Fedele and Brand 2020); in pigs, stellate ganglia cell firing is modulated by apnea on the order of seconds (Sudarshan et al. 2021), indicating rapid sympathetic engagement by reflexive pathways. In some cases, we observed saw-tooth patterns of instantaneous HR in response to burst patterns of VNS with high MPR (e.g., 100 Hz intra-burst frequency, 50 Hz MPR) and post-stimulation HR tachycardia (HR_norm_ > 1). The HR model, which did not include reflexive sympathetic mechanisms, did not produce such HR dynamics in simulations of identical stimulation patterns (Additional file 1: Fig. 23). Further analysis of time-dependent HR dynamics may elucidate the time-course of HR recovery after VNS-induced bradycardia via sympathetic mechanisms.

Phenomenological models of VNS effects on HR can replicate a range of cardiac responses to VNS (e.g., (Haberbusch et al. 2022)). However, such models cannot leverage biological mechanisms to predict stimulation outcomes that were not used to parameterize the model. For example, a biologically informed model of VNS may predict bradycardia-induced arrhythmias through inclusion of myocardia conduction (e.g., the SAN network) whereas the phenomenological models would not unless data of arrhythmias were used for model training. We did not observe arrhythmias in our SAN network model, consistent with reports of electrical stability of mouse cardiac tissue; transgenic approaches may be necessary to reproduce arrhythmias (Choy et al. 2016; Dobrev and Wehrens 2018).

Our parameterized model of muscle force matched in vivo findings but required several assumptions. Direct measurement of laryngeal muscle force was infeasible for model validation and comparisons. Rather, we relied on a linear relationship between EMG and force, which has been demonstrated in patients with functional electrical stimulation (FES) (Mizrahi et al. 1997) and simulations of synchronized motor unit activity (Zhou and Rymer 2004). With this assumption, we found strong agreement between in vivo and modeled muscle activation, although these results could be further validated through modeling EMG and directly comparing model outcomes with in vivo measurements (Petersen and Rostalski 2019). We assumed that muscle activation as quantified by EMG was an appropriate metric for side effects of VNS experienced by patients. Parametric studies with patients using VNS could verify patient discomfort correlates with muscle activation as measured by EMG. Several muscle model parameters reached value constraints defined by published studies of human quadriceps femoris force production. While we observed good agreement in modeled and measured muscle activation, further investigation of model constraints and optimization is warranted.

The agreement in effect score between in vivo measurements and model outcomes supports the utility of the computational models to quantify therapy and side effects during stimulation of a compound nerve. Translation will likely require clinical data on human physiological response to parameter variation for human-specific (or patient-specific) model construction due to anatomical and physiological differences in laryngeal muscles and parasympathetic innervation to the heart. Both models presented here were specific to mouse physiology which differs substantially from humans; resting HR in anesthetized mice is roughly 6-fold that in human (400 BPM and 60 BPM, respectively). Single cell models of human SAN pacemaker cells have been validated (Fabbri et al. 2017; Pohl et al. 2016), but modeling of VNS-evoked changes in HR requires parametric studies. Muscle fiber composition profoundly influences muscle contraction and fatigue characteristics, and human laryngeal muscles have higher proportion of slow-twitch, fatigue-resistant fibers than in mice (Hoh 2005). This implies the fatigue response observed at the higher frequencies may not apply in humans. While VNS-evoked EMG has been collected in humans (Saibene et al. 2017), larger parametric tests of VNS-evoked laryngeal muscle activation are required for model parameterization and validation.

Nerve size and morphology should also be considered as the mouse CVN is ~ 150 μm in diameter and monofascicular whereas the human CVN is ~ 2 mm in diameter and subdivided into fascicles (Pelot et al. 2020; Stakenborg et al. 2020). This discrepancy influences the nerve response to VNS and, in humans, may allow for spatial selectivity. While the number of fibers in the human CVN is more than 30-fold greater than in the mouse CVN, the fiber population proportions are strikingly similar (Stakenborg et al. 2020). Taken together, the physiological differences between mice and humans could influence our finding that 1.2xBCT at 40 Hz increased HR effects over laryngeal muscle activation.

For the primary range of stimulation amplitudes (0.8-1.2xBCT), we theorized the activation of large, myelinated A fibers that mediated laryngeal muscle activity was saturated (or close to saturated); conversely, those stimulation amplitudes were within the dynamic range of the smaller, thinly myelinated preganglionic efferent B fibers that mediated bradycardia (Yoo et al. 2016). Subsequent experiments with an expanded stimulation amplitude range (0.2-2.0xBCT; BCT = 0.035-0.12 mA) revealed an overlap in the dynamic ranges of HR and EMG outcomes (i.e., activation of A and B fibers). We observed slight EMG waveform changes at stimulation amplitudes above 1.2xBCT (Fig. 11B) that may indicate excitation of fibers outside of the cuff (i.e., superior laryngeal branch of the vagus nerve) via current leakage as observed in pigs (Nicolai et al. 2020) and may explain EMG_norm_ values substantially greater than 1 (e.g., LV4 and WI7). Additionally, the computational model illustrated that the longitudinal alignment of the nodes of Ranvier of A fibers, but not B fibers, strongly affected recruitment order and produced overlap between the excitation ranges of A fibers and B fibers, despite the smaller diameters of the B fibers.

The FEM and biophysical cable model predicted response to stimulation amplitude had good agreement for most EMG responses but not HR responses. The model captured onset threshold for A fibers (identified by EMG) for some animals (e.g., animals AA1 and DP8) but underestimated thresholds for others (e.g., animals LV4 and AB1). For all animals, the model underestimated onset threshold for B fibers (i.e., BCT, identified by HR changes), and the HR dynamic range extended beyond the highest B fiber thresholds. Discrepancy between modeled and in vivo nerve responses can be attributed to sources of experimental variability and to model limitations. High EMG onset and BCT amplitudes may be caused by inter-experimental variability such as air bubbles between the electrode contact and the nerve. Model threshold accuracy depended on fiber type where the same electric field was applied to all biophysical cable models, indicating fiber model choice may contribute to the difference between B fiber threshold and bradycardia onset. Our biophysical cable models of B fibers were scaled MRG (McIntyre-Richardson-Grill) myelinated fiber models (McIntyre et al. 2002). While the MRG model reproduces excitation properties of A fibers, the suitability to model thinly myelinated parasympathetic efferent vagal fibers warrants further investigation.

## Conclusions

The widespread innervation by the vagus presents opportunities to use VNS across a range of applications (Guiraud et al. 2016) but also increases the potential for myriad off-target effects. Future efforts should focus on modeling VNS effects on other organs, CNS nuclei, and connected brain regions (Borovikova et al. 2000; Tsaava et al. 2020). For example, modeling vagal innervation of the spleen would require data of release dynamics of norepinephrine from splenic nerve fibers, the rate of ACh production by T-cells, and inhibition of cytokine release from muscarinic receptor-expressing macrophages. No step of this process has been studied with the temporal fidelity required for a computational model, but such a model would allow increased efficacy in VNS for treatment of sepsis and autoimmune disorders (Kelly et al. 2022). In conclusion, we demonstrated HR and laryngeal muscle activation respond differently to VNS parameter selection and accurate computational models provide insight into the physiological factors that dictate this response.

## Supplementary Information

**Additional file 1: Figure 12.** Phenomenological function of success rate of vagal action potential to result in post-ganglionic action potential. Data (black dots) extracted from (McAllen et al. 2011) (black points) fit to exponential curve (red line) where *t* is the time of a vagal action potential and *t*_0_ is the time of the previous post-ganglionic action potential in ms. **Figure 13.** Phasic activation of intrinsic cardiac nervous system (ICNS) cells. A) Measured excitatory post-synaptic potentials (EPSP, grey bars) and overlaid normalized phrenic nerve activity (black line) in a working rat heart-brainstem preparation. Figure adapted with permission from (McAllen et al. 2011). B) Illustrative raster plot of 100 modeled cycles of ICNS firing in 1.3 s epoch. C) Associated cycle-triggered event histogram of data from (B) to compare with (A). **Figure 14.** Validation of implementation of acetylcholine (ACh) release using 3 compartment model. Model implementation (black line) was validated using published data from (Dokos et al. 1996a) (red points) for three ACh hydrolysis time constants (rows; *k*_H_, 14 s^− 1^, 5 s^− 1^, 30 s^− 1^) and two vagus nerve stimulation frequencies (10 Hz and 100 Hz). **Figure 15.** Validation of Kharche model of sinoatrial node cell firing (Kharche et al. 2011). Model implementation in Python + NEURON (black lines) overlaid with data extracted from published figures (dashed red lines) and published model in MATLAB from (Morotti et al. 2021). A) Transmembrane potential. B-M) Transmembrane ion channel currents (I, pA/pF). Differences between data from published figures and model implementations in C and L may arise from plotting errors in the original publication. N-P) Intracellular ion concentrations. **Figure 16.** Validation of Ding model of muscle force production and fatigue by comparison of model implementation (black lines) to published data (orange circles). Model parameters and validation data from (Ding et al. 2003). Simulation is of force production of the quadriceps femoris in a healthy individual undergoing electrical stimulation (30 Hz, 1.5 s on, 0.5 s off). A) Force production for first 10 s of simulation (left) and after extended period of constant frequency stimulation (right). A decrease in evoked force is indicative of muscle fatigue. B-D) Time series of dynamic changes of model parameters shows that model implementation and published data agree. **Figure 17.** Range of cost function values (grey area) and lowest cost function value (black line) for particle swarm optimization (PSO) parameterization of computational model of VNS-evoked laryngeal force. A) Five PSO runs varied across initial particle positions and training/testing sets. The PSO run that produced the model parameters with the lowest combined training and testing error is identified by gold area and red line. This run did not produce the lowest “testing” Cost Function value. B) Reproduction of combination of training and testing sets that produced the optimal parameters with varied particle initial positions. **Table 4.** Normalized heart rate (HR_norm_) outcomes and post-hoc test comparing means to 1.0xBCT, 20 Hz. **Table 5.** Normalized EMG (EMG_norm_) and post-hoc test comparing means to 1.0xBCT, 20 Hz. **Table 6.** Effect score outcomes and post-hoc test comparing means to 1.0xBCT, 20 Hz. **Figure 18.** Effect on HR_norm_ of stimulation patterns with 0.5 s stim, 0.5 s pause (50% duty cycle). A) Stimulation patterns. B) HR normalized to pre-stimulation baseline (HR_norm_) and plotted across mean pulse rate (x-axis) and amplitude (color). Data are presented as mean ± SE, *n* = 8-10/parameter set. **Figure 19.** Correlation between in vivo physiological responses and frequency characteristics of random patterns of VNS. A-B) No correlation detected between HR and mean inter-pulse frequency (<IPF>, A) or geometric <IPF> (<IPF > _geo_, B) using linear fits. C-D) No correlation detected between EMG and < IPF> (C) or < IPF>_geo_ (D). Data points are outcomes from individual experiments (*n* = 10 per MPR value). Coefficient of determination (R^2^) from linear fits. No fit was statistically different from constant values (*p* > 0.05). **Figure 20.** Comparisons of simulated and in vivo data for HRnorm (A), muscle activation (B, EMG_norm_ for in vivo data, Force_norm_ for model data), and effect score (C). Comparison of modeled and in vivo HR_norm_ and effect score performed at 0.8xBCT (i), 1.0xBCT (ii), and 1.2xBCT (iii). EMG_norm_ and Force_norm_ compared for 1.0xBCT. Data are presented as mean ± SE, *n* = 8-10/parameter set for in vivo data, *n* = 10 runs per parameter set for computational models. **Table 7.** Error between modeled normalized heart rate (HR_norm, model_) and in vivo HR_norm,in vivo_. **Table 8.** Error between modeled normalized force (Force_norm_) and in vivo normalized EMG (EMG_norm_). **Table 9.** Error between modeled effect score (Effect Score_model_) and in vivo effect score (Effect Score_in vivo_). **Figure 21.** Recruitment curves for modeled A and B fibers colored to indicate fiber “jitter” (i.e., longitudinal shift of fibers as a proportion of internodal length) overlaid with in vivo EMG (A) and normalized heart rate (B) responses in five animals across stimulation amplitudes. **Figure 22.** EMG waveform depression during high-frequency VNS. A) EMG recordings of two trials within the same animal (XD0). EMG waveform amplitude stays consistent during 5 Hz stimulation B). Evoked EMG waveform amplitude is similar at beginning of the trial (i) and is greatly reduced at the end of the trial during 100 Hz stimulation. **Figure 23.** Comparison of dynamic normalized heart rate (HR_norm_) values for representative in vivo trial (animal HU0, solid line) and simulation (dashed line) during 1.0xBCT, 100 Hz intra-burst frequency, and 50 MPR stimulation.

## Data Availability

All data and code (in vivo data, model data, model code) are publicly available and cited in the paper.

## Abbreviations

ACh: Acetylcholine
BCT: Bradycardia threshold
BPM: Beats per minute
CVN: Cervical vagus nerve
ECG: Electrocardiogram
EMG: Electromyogram
EMG_arv_: EMG average rectified value
EMG_norm_: Normalized EMG
FES: Functional electrical stimulation
FF: Fast fatigable
Force_norm_: Normalized force
FR: Fatigue resistant
HR: Heart failure
HR: Heart rate
HR_baseline_: Baseline HR
HR_norm_: Normalized HR
HR_stim_: Stimulation HR
ICNS: Intrinsic cardiac nervous system
IPF: Intra-pulse frequency
MPR: Mean pulse rate
MU: Motor unit
PSO: Particle swarm optimization
S: Slow
SS: Sum of squares
V_m_: Transmembrane voltage
VN: Vagus nerve
VNS: Vagus nerve stimulation
VNX: Vagotomy
