# Supplementary material for: Measuring and modeling the effects of vagus nerve stimulation on heart rate and laryngeal muscles
Source: Bioelectron Med. 2023 Feb 17;9:3. doi: 10.1186/s42234-023-00107-4 (PMC9936668; doi:10.1186/s42234-023-00107-4)
Supplement: Supplementary file 1 — Additional file 1: Figure 12. Phenomenological function of success rate of vagal action potential to result in post-ganglionic action potential. Data (black dots) extracted from (McAllen et al. 2011) (black points) fit to exponential curve (red line) where t is the time of a vagal action potential and t0 is the time of the previous post-ganglionic action potential in ms. Figure 13. Phasic activation of intrinsic cardiac nervous system (ICNS) cells. A) Measured excitatory post-synaptic potentials (EPSP, grey bars) and overlaid normalized phrenic nerve activity (black line) in a working rat heart-brainstem preparation. Figure adapted with permission from (McAllen et al. 2011). B) Illustrative raster plot of 100 modeled cycles of ICNS firing in 1.3 s epoch. C) Associated cycle-triggered event histogram of data from (B) to compare with (A). Figure 14. Validation of implementation of acetylcholine (ACh) release using 3 compartment model. Model implementation (black line) was validated using published data from (Dokos et al. 1996a) (red points) for three ACh hydrolysis time constants (rows; kH, 14 s− 1, 5 s− 1, 30 s− 1) and two vagus nerve stimulation frequencies (10 Hz and 100 Hz). Figure 15. Validation of Kharche model of sinoatrial node cell firing (Kharche et al. 2011). Model implementation in Python + NEURON (black lines) overlaid with data extracted from published figures (dashed red lines) and published model in MATLAB from (Morotti et al. 2021). A) Transmembrane potential. B-M) Transmembrane ion channel currents (I, pA/pF). Differences between data from published figures and model implementations in C and L may arise from plotting errors in the original publication. N-P) Intracellular ion concentrations. Figure 16. Validation of Ding model of muscle force production and fatigue by comparison of model implementation (black lines) to published data (orange circles). Model parameters and validation data from (Ding et al. 2003). Simulation is of force production of th [file 42234_2023_107_MOESM1_ESM.docx]

Supplementary Materials


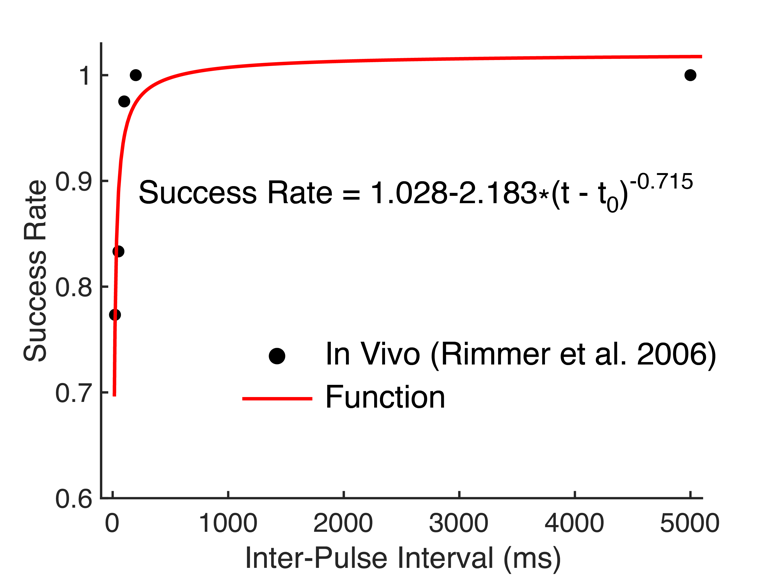


Figure 12. Phenomenological function of success rate of vagal action potential to result in post-ganglionic action potential. Data (black dots) extracted from (McAllen et al., 2011) (black points) fit to exponential curve (red line) where $t$ is the time of a vagal action potential and $t_{0}$ is the time of the previous post-ganglionic action potential in ms.


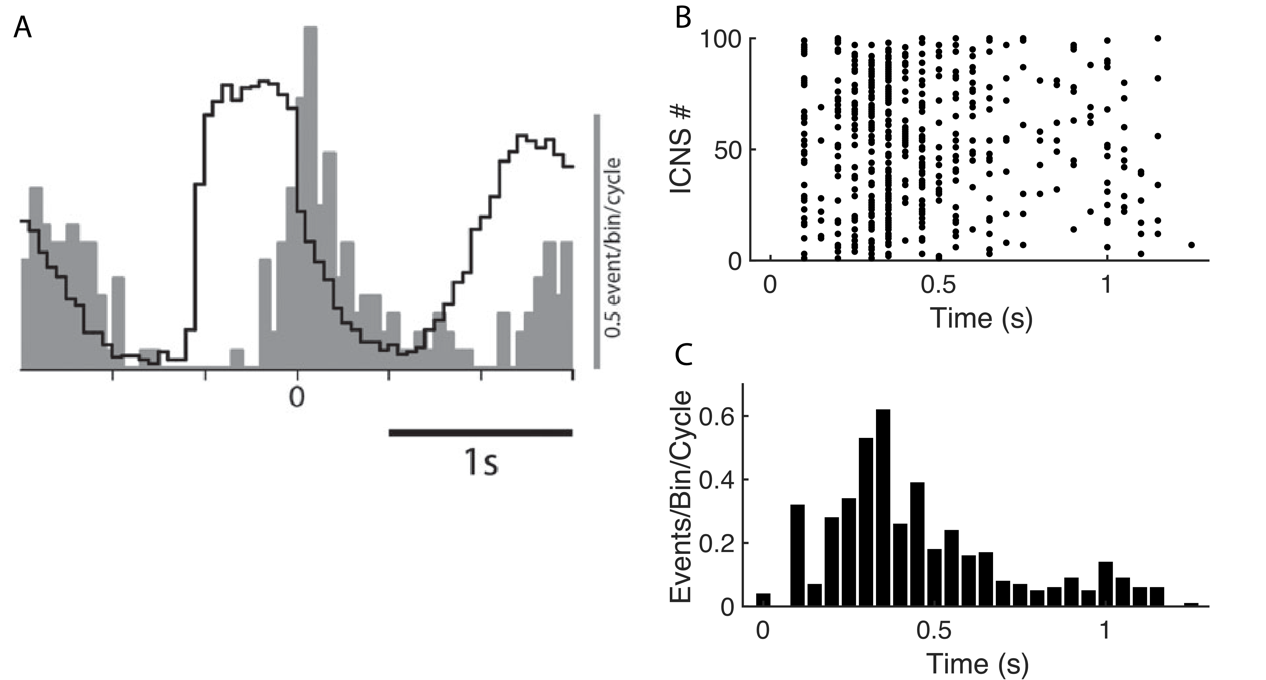


Figure 13. Phasic activation of intrinsic cardiac nervous system (ICNS) cells. A) Measured excitatory post-synaptic potentials (EPSP, grey bars) and overlaid normalized phrenic nerve activity (black line) in a working rat heart-brainstem preparation. Figure adapted with permission from (McAllen et al., 2011). B) Illustrative raster plot of 100 modeled cycles of ICNS firing in 1.3 s epoch. C) Associated cycle-triggered event histogram of data from (B) to compare with (A).


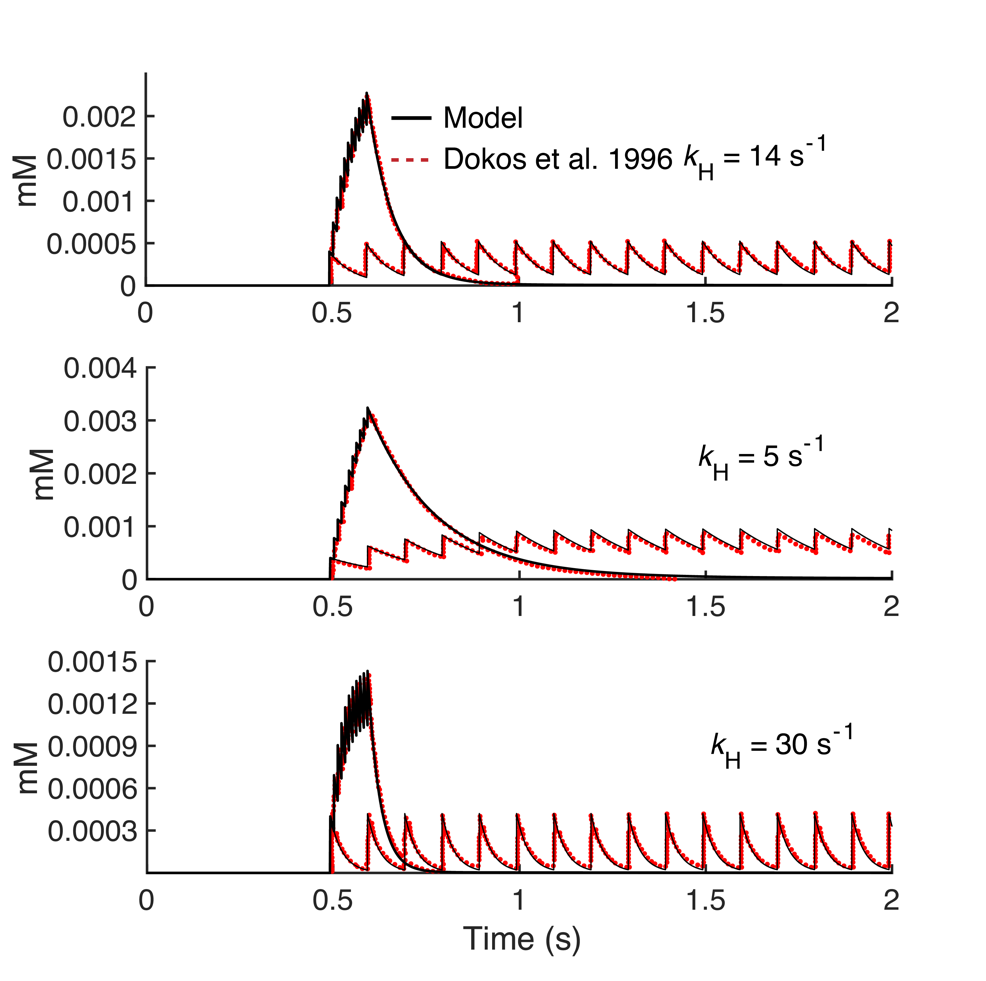


Figure 14. Validation of implementation of acetylcholine (ACh) release using 3 compartment model. Model implementation (black line) was validated using published data from (Dokos et al., 1996a) (red points) for three ACh hydrolysis time constants (rows; $k_{H}$, 14 s^-1^, 5 s^-1^, 30 s^-1^) and two vagus nerve stimulation frequencies (10 Hz and 100 Hz).


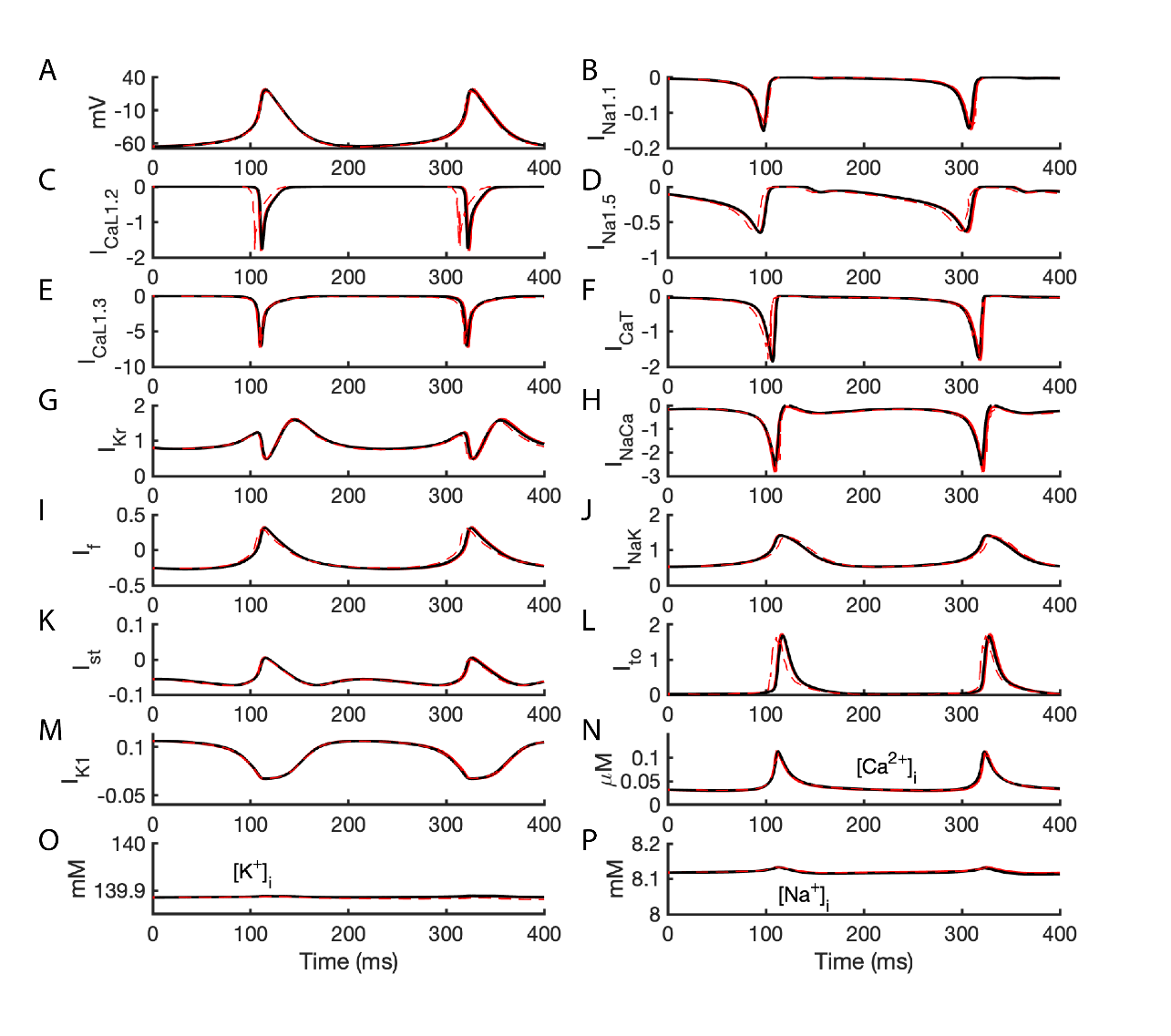


Figure 15. Validation of Kharche model of sinoatrial node cell firing (Kharche et al., 2011). Model implementation in Python + NEURON (black lines) overlaid with data extracted from published figures (dashed red lines) and published model in MATLAB from (Morotti et al., 2021). A) Transmembrane potential. B-M) Transmembrane ion channel currents (I, pA/pF). Differences between data from published figures and model implementations in C and L may arise from plotting errors in the original publication. N-P) Intracellular ion concentrations.


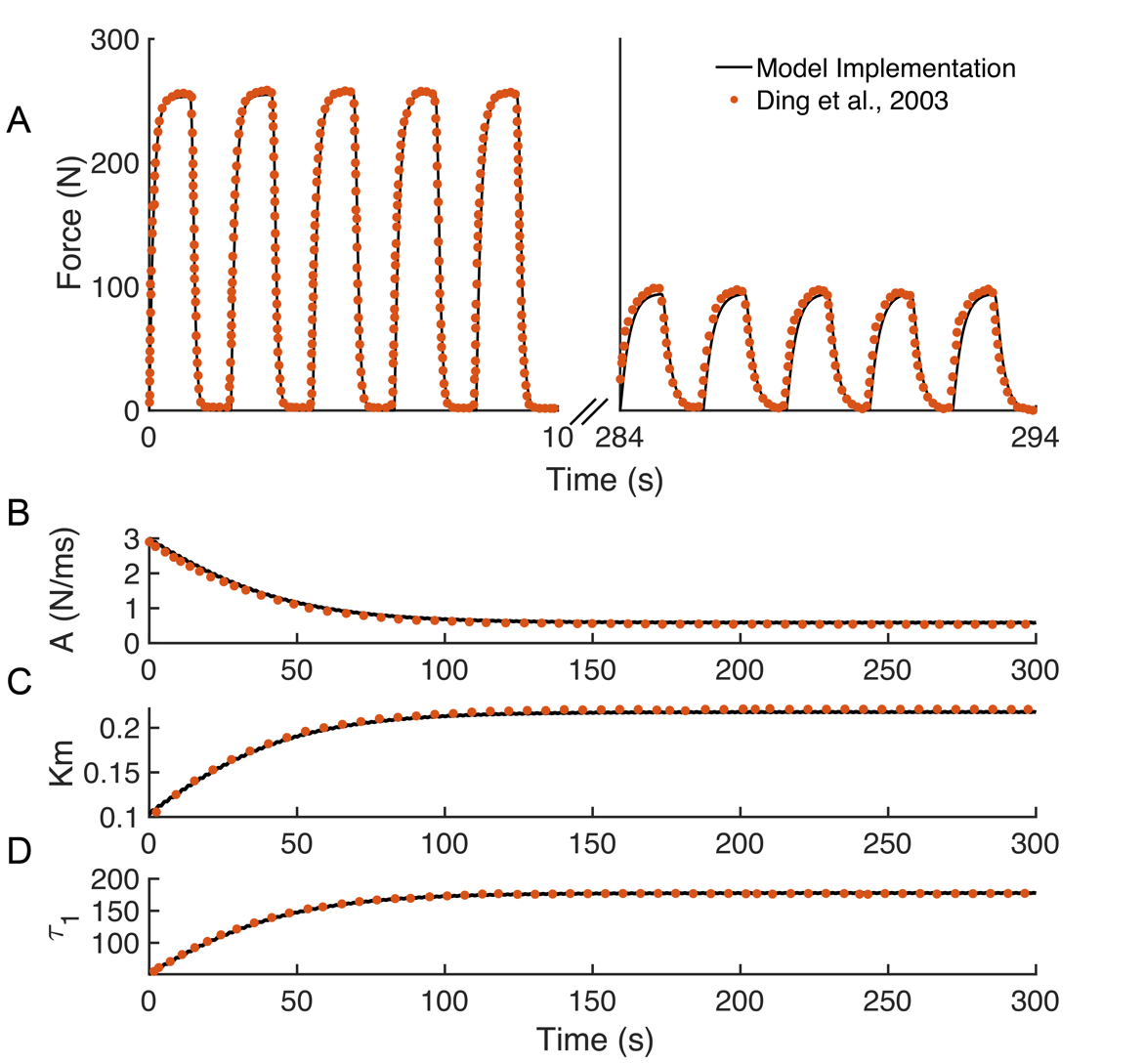


Figure 16. Validation of Ding model of muscle force production and fatigue by comparison of model implementation (black lines) to published data (orange circles). Model parameters and validation data from (Ding et al., 2003). Simulation is of force production of the quadriceps femoris in a healthy individual undergoing electrical stimulation (30 Hz, 1.5 s on, 0.5 s off). A) Force production for first 10 s of simulation (left) and after extended period of constant frequency stimulation (right). A decrease in evoked force is indicative of muscle fatigue. B-D) Time series of dynamic changes of model parameters shows that model implementation and published data agree.


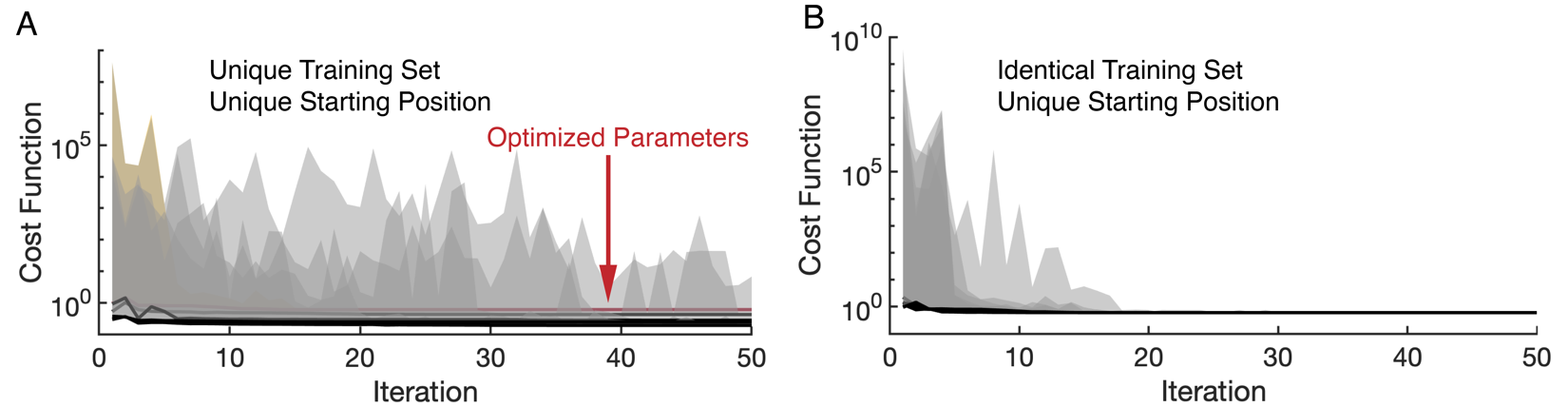


Figure 17. Range of cost function values (grey area) and lowest cost function value (black line) for particle swarm optimization (PSO) parameterization of computational model of VNS-evoked laryngeal force. A) Five PSO runs varied across initial particle positions and training/testing sets. The PSO run that produced the model parameters with the lowest combined training and testing error is identified by gold area and red line. This run did not produce the lowest “testing” Cost Function value. B) Reproduction of combination of training and testing sets that produced the optimal parameters with varied particle initial positions.

Table 4. Normalized heart rate (HR_norm_) outcomes and post-hoc test comparing means to 1.0xBCT, 20 Hz.

| HR_norm_  mean +/- standard deviation | | | Mean Pulse Rate | | | | | | | |
| --- | --- | --- | --- | --- | --- | --- | --- | --- | --- | --- |
|  |  |  | 2 | 5 | 10 | 20 | 30 | 40 | 50 | 100 |
| 0.8xBCT | Burst Frequency (Hz) | 2 | 0.99 +/- 0.01 |  |  |  |  |  |  |  |
|  |  | 5 | 0.99 +/- 0.01 | 0.98 +/- 0.02 |  |  |  |  |  |  |
|  |  | 10 | 0.99 +/- 0.01 | 0.98 +/- 0.02 | 0.96 +/- 0.03 |  |  |  |  |  |
|  |  | 20 | 0.98 +/- 0.02 | 0.97 +/- 0.03 | 0.98 +/- 0.02 | 0.96 +/- 0.02 |  |  |  |  |
|  |  | 30 | 1.00 +/- 0.01 | 0.98 +/- 0.01 | 0.98 +/- 0.02 | 0.96 +/- 0.03 | 0.96 +/- 0.04 |  |  |  |
|  |  | 40 | 0.99 +/- 0.02 | 0.98 +/- 0.02 | 0.97 +/- 0.04 | 0.98 +/- 0.04 | 0.95 +/- 0.05 | 0.92 +/- 0.08 |  |  |
|  |  | 50 | 0.99 +/- 0.01 | 0.99 +/- 0.03 | 0.98 +/- 0.03 | 0.95 +/- 0.02 | 0.96 +/- 0.02 | 0.93 +/- 0.05 | 0.88 +/- 0.13 |  |
|  |  | 100 | 1.00 +/- 0.01 | 0.98 +/- 0.03 | 0.97 +/- 0.03 | 0.96 +/- 0.06 | 0.91 +/- 0.08 | 0.91 +/- 0.09 | 0.95 +/- 0.05 | 0.81 +/- 0.14 |
|  | | | | | | | | | | |
| 1.0xBCT | Burst Frequency (Hz) | 2 | 0.98 +/- 0.02 |  |  |  |  |  |  |  |
|  |  | 5 | 0.98 +/- 0.01 | 0.97 +/- 0.02 |  |  |  |  |  |  |
|  |  | 10 | 0.98 +/- 0.02 | 0.97 +/- 0.02 | 0.93 +/- 0.06 |  |  |  |  |  |
|  |  | 20 | 0.97 +/- 0.03 | 0.97 +/- 0.02 | 0.94 +/- 0.03 | **0.84 +/- 0.07** |  |  |  |  |
|  |  | 30 | 0.99 +/- 0.02 | 0.95 +/- 0.04 | 0.95 +/- 0.03 | 0.88 +/- 0.07 | 0.85 +/- 0.08 |  |  |  |
|  |  | 40 | 0.96 +/- 0.04 | 0.97 +/- 0.03 | 0.94 +/- 0.03 | 0.87 +/- 0.10 | 0.88 +/- 0.12 | 0.71 +/- 0.15 |  |  |
|  |  | 50 | 0.98 +/- 0.01 | 0.97 +/- 0.01 | 0.94 +/- 0.03 | 0.91 +/- 0.05 | 0.83 +/- 0.09 | 0.83 +/- 0.11 | 0.63 +/- 0.15 |  |
|  |  | 100 | 0.98 +/- 0.02 | 0.96 +/- 0.04 | 0.94 +/- 0.05 | 0.89 +/- 0.06 | 0.85 +/- 0.12 | 0.85 +/- 0.12 | 0.78 +/- 0.16 | 0.60 +/- 0.19 |
|  | | | | | | | | | | |
| 1.2xBCT | Burst Frequency (Hz) | 2 | 0.97 +/- 0.03 |  |  |  |  |  |  |  |
|  |  | 5 | 0.98 +/- 0.02 | 0.94 +/- 0.04 |  |  |  |  |  |  |
|  |  | 10 | 0.97 +/- 0.02 | 0.93 +/- 0.05 | 0.91 +/- 0.05 |  |  |  |  |  |
|  |  | 20 | 0.97 +/- 0.02 | 0.95 +/- 0.02 | 0.90 +/- 0.05 | 0.83 +/- 0.05 |  |  |  |  |
|  |  | 30 | 0.98 +/- 0.01 | 0.94 +/- 0.03 | 0.87 +/- 0.07 | 0.76 +/- 0.11 | 0.65 +/- 0.13 |  |  |  |
|  |  | 40 | 0.97 +/- 0.03 | 0.95 +/- 0.03 | 0.87 +/- 0.06 | 0.76 +/- 0.11 | 0.60 +/- 0.14 | 0.57 +/- 0.17 |  |  |
|  |  | 50 | 0.97 +/- 0.02 | 0.94 +/- 0.03 | 0.87 +/- 0.07 | 0.76 +/- 0.10 | 0.67 +/- 0.15 | 0.68 +/- 0.14 | 0.54 +/- 0.23 |  |
|  |  | 100 | 0.98 +/- 0.01 | 0.93 +/- 0.04 | 0.90 +/- 0.05 | 0.78 +/- 0.03 | 0.74 +/- 0.12 | 0.65 +/- 0.17 | 0.72 +/- 0.14 | 0.48 +/- 0.23 |
| p<0.05 | |  | | | | | | | | |

Table 5. Normalized EMG (EMG_norm_) and post-hoc test comparing means to 1.0xBCT, 20 Hz.

| EMG_norm_  mean +/- standard deviation | | | Mean Pulse Rate | | | | | | | |
| --- | --- | --- | --- | --- | --- | --- | --- | --- | --- | --- |
|  |  |  | 2 | 5 | 10 | 20 | 30 | 40 | 50 | 100 |
| 0.8xBCT | Burst Frequency (Hz) | 2 | 0.08 +/- 0.02 |  |  |  |  |  |  |  |
|  |  | 5 | 0.08 +/- 0.04 | 0.21 +/- 0.06 |  |  |  |  |  |  |
|  |  | 10 | 0.09 +/- 0.03 | 0.21 +/- 0.07 | 0.40 +/- 0.11 |  |  |  |  |  |
|  |  | 20 | 0.08 +/- 0.02 | 0.20 +/- 0.04 | 0.45 +/- 0.08 | 0.85 +/- 0.23 |  |  |  |  |
|  |  | 30 | 0.09 +/- 0.04 | 0.22 +/- 0.04 | 0.44 +/- 0.19 | 0.98 +/- 0.22 | 1.41 +/- 0.39 |  |  |  |
|  |  | 40 | 0.08 +/- 0.02 | 0.20 +/- 0.04 | 0.39 +/- 0.09 | 0.88 +/- 0.15 | 1.42 +/- 0.36 | 1.72 +/- 0.36 |  |  |
|  |  | 50 | 0.07 +/- 0.04 | 0.23 +/- 0.09 | 0.45 +/- 0.16 | 0.73 +/- 0.17 | 1.17 +/- 0.26 | 1.63 +/- 0.29 | 1.98 +/- 0.52 |  |
|  |  | 100 | 0.08 +/- 0.02 | 0.19 +/- 0.05 | 0.41 +/- 0.17 | 0.77 +/- 0.16 | 1.05 +/- 0.36 | 1.62 +/- 0.29 | 2.07 +/- 0.66 | 3.16 +/- 0.73 |
|  | | | | | | | | | | |
| 1.0xBCT | Burst Frequency (Hz) | 2 | 0.09 +/- 0.02 |  |  |  |  |  |  |  |
|  |  | 5 | 0.10 +/- 0.02 | 0.24 +/- 0.05 |  |  |  |  |  |  |
|  |  | 10 | 0.13 +/- 0.10 | 0.24 +/- 0.05 | 0.51 +/- 0.12 |  |  |  |  |  |
|  |  | 20 | 0.11 +/- 0.03 | 0.24 +/- 0.08 | 0.52 +/- 0.17 | **1.00** |  |  |  |  |
|  |  | 30 | 0.09 +/- 0.02 | 0.27 +/- 0.05 | 0.55 +/- 0.10 | 0.98 +/- 0.22 | 1.52 +/- 0.41 |  |  |  |
|  |  | 40 | 0.10 +/- 0.02 | 0.26 +/- 0.05 | 0.52 +/- 0.08 | 1.01 +/- 0.20 | 1.26 +/- 0.25 | 1.62 +/- 0.33 |  |  |
|  |  | 50 | 0.09 +/- 0.02 | 0.22 +/- 0.05 | 0.43 +/- 0.08 | 0.86 +/- 0.17 | 1.20 +/- 0.13 | 1.67 +/- 0.33 | 2.04 +/- 0.50 |  |
|  |  | 100 | 0.09 +/- 0.02 | 0.23 +/- 0.06 | 0.45 +/- 0.12 | 0.85 +/- 0.12 | 1.25 +/- 0.27 | 1.79 +/- 0.35 | 2.06 +/- 0.68 | 2.92 +/- 0.71 |
|  | | | | | | | | | | |
| 1.2xBCT | Burst Frequency (Hz) | 2 | 0.11 +/- 0.04 |  |  |  |  |  |  |  |
|  |  | 5 | 0.11 +/- 0.03 | 0.28 +/- 0.07 |  |  |  |  |  |  |
|  |  | 10 | 0.11 +/- 0.03 | 0.27 +/- 0.07 | 0.50 +/- 0.12 |  |  |  |  |  |
|  |  | 20 | 0.12 +/- 0.06 | 0.27 +/- 0.07 | 0.52 +/- 0.11 | 1.10 +/- 0.33 |  |  |  |  |
|  |  | 30 | 0.10 +/- 0.03 | 0.29 +/- 0.07 | 0.59 +/- 0.12 | 1.22 +/- 0.34 | 1.61 +/- 0.35 |  |  |  |
|  |  | 40 | 0.11 +/- 0.02 | 0.27 +/- 0.07 | 0.51 +/- 0.16 | 1.11 +/- 0.30 | 1.50 +/- 0.20 | 1.73 +/- 0.16 |  |  |
|  |  | 50 | 0.10 +/- 0.02 | 0.27 +/- 0.06 | 0.49 +/- 0.06 | 0.96 +/- 0.18 | 1.30 +/- 0.15 | 1.90 +/- 0.55 | 2.54 +/- 0.77 |  |
|  |  | 100 | 0.10 +/- 0.03 | 0.26 +/- 0.05 | 0.43 +/- 0.11 | 0.87 +/- 0.23 | 1.23 +/- 0.25 | 1.75 +/- 0.53 | 1.95 +/- 0.30 | 3.27 +/- 1.92 |
| p<0.05 | |  | | | | | | | | |

Table 6. Effect score outcomes and post-hoc test comparing means to 1.0xBCT, 20 Hz.

| Effect Score  mean +/- standard deviation | | | Mean Pulse Rate | | | | | | | |
| --- | --- | --- | --- | --- | --- | --- | --- | --- | --- | --- |
|  |  |  | 2 | 5 | 10 | 20 | 30 | 40 | 50 | 100 |
| 0.8xBCT | Burst Frequency (Hz) | 2 | -0.02 +/- 0.11 |  |  |  |  |  |  |  |
|  |  | 5 | 0.00 +/- 0.12 | -0.07 +/- 0.21 |  |  |  |  |  |  |
|  |  | 10 | 0.01 +/- 0.11 | -0.02 +/- 0.38 | -0.11 +/- 0.26 |  |  |  |  |  |
|  |  | 20 | 0.06 +/- 0.16 | 0.05 +/- 0.24 | -0.30 +/- 0.22 | -0.50 +/- 0.58 |  |  |  |  |
|  |  | 30 | -0.09 +/- 0.08 | -0.11 +/- 0.07 | -0.31 +/- 0.26 | -0.72 +/- 0.29 | -1.16 +/- 0.60 |  |  |  |
|  |  | 40 | 0.01 +/- 0.15 | -0.02 +/- 0.14 | -0.19 +/- 0.19 | -0.63 +/- 0.27 | -1.24 +/- 0.47 | -1.01 +/- 0.67 |  |  |
|  |  | 50 | 0.00 +/- 0.13 | -0.15 +/- 0.29 | -0.29 +/- 0.33 | -0.24 +/- 0.57 | -0.75 +/- 0.41 | -1.07 +/- 0.70 | -0.88 +/- 0.76 |  |
|  |  | 100 | -0.05 +/- 0.06 | 0.02 +/- 0.36 | -0.19 +/- 0.12 | -0.45 +/- 0.36 | -0.41 +/- 1.11 | -1.02 +/- 0.57 | -1.72 +/- 0.67 | -1.63 +/- 1.05 |
|  | | | | | | | | | | |
| 1.0xBCT | Burst Frequency (Hz) | 2 | 0.05 +/- 0.13 |  |  |  |  |  |  |  |
|  |  | 5 | 0.03 +/- 0.10 | 0.02 +/- 0.25 |  |  |  |  |  |  |
|  |  | 10 | 0.03 +/- 0.20 | -0.03 +/- 0.18 | -0.03 +/- 0.33 |  |  |  |  |  |
|  |  | 20 | 0.13 +/- 0.24 | -0.01 +/- 0.27 | 0.00 +/- 0.45 | **0.00** |  |  |  |  |
|  |  | 30 | 0.01 +/- 0.09 | 0.12 +/- 0.37 | -0.09 +/- 0.36 | 0.08 +/- 1.25 | -0.28 +/- 0.78 |  |  |  |
|  |  | 40 | 0.08 +/- 0.17 | -0.04 +/- 0.31 | -0.06 +/- 0.34 | -0.03 +/- 0.68 | -0.34 +/- 0.72 | 0.43 +/- 1.09 |  |  |
|  |  | 50 | 0.03 +/- 0.13 | 0.04 +/- 0.23 | 0.09 +/- 0.47 | -0.08 +/- 0.83 | -0.03 +/- 0.80 | -0.29 +/- 0.65 | 0.78 +/- 1.53 |  |
|  |  | 100 | 0.04 +/- 0.19 | 0.15 +/- 0.49 | -0.03 +/- 0.43 | 0.02 +/- 0.68 | -0.25 +/- 0.61 | -0.58 +/- 1.07 | -0.41 +/- 1.30 | 0.07 +/- 2.39 |
|  | | | | | | | | | | |
| 1.2xBCT | Burst Frequency (Hz) | 2 | 0.03 +/- 0.09 |  |  |  |  |  |  |  |
|  |  | 5 | 0.01 +/- 0.11 | 0.11 +/- 0.23 |  |  |  |  |  |  |
|  |  | 10 | 0.13 +/- 0.12 | 0.22 +/- 0.28 | 0.25 +/- 0.60 |  |  |  |  |  |
|  |  | 20 | 0.13 +/- 0.21 | 0.11 +/- 0.22 | 0.20 +/- 0.42 | 0.10 +/- 0.75 |  |  |  |  |
|  |  | 30 | 0.06 +/- 0.13 | 0.22 +/- 0.46 | 0.31 +/- 0.44 | 0.50 +/- 0.94 | 0.96 +/- 1.16 |  |  |  |
|  |  | 40 | 0.11 +/- 0.18 | 0.17 +/- 0.47 | 0.41 +/- 0.54 | 0.44 +/- 0.53 | 1.28 +/- 0.97 | 1.82 +/- 2.14 |  |  |
|  |  | 50 | 0.13 +/- 0.14 | 0.17 +/- 0.23 | 0.34 +/- 0.41 | 0.65 +/- 0.68 | 0.95 +/- 0.97 | 0.68 +/- 1.45 | 1.14 +/- 1.61 |  |
|  |  | 100 | 0.02 +/- 0.08 | 0.21 +/- 0.30 | 0.28 +/- 0.54 | 0.82 +/- 1.14 | 0.83 +/- 1.42 | 0.94 +/- 1.86 | 0.23 +/- 1.29 | 0.47 +/- 3.28 |
| p<0.05 | |  | | | | | | | | |


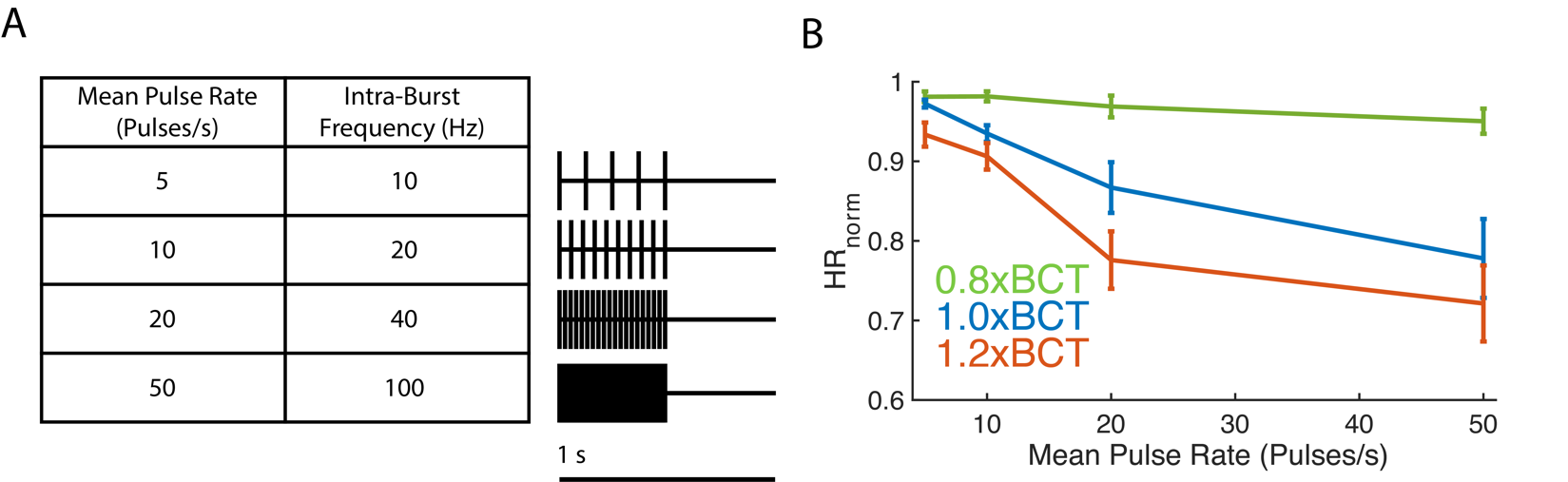


Figure 18. Effect on HR_norm_ of stimulation patterns with 0.5 s stim, 0.5 s pause (50% duty cycle). A) Stimulation patterns. B) HR normalized to pre-stimulation baseline (HR_norm_) and plotted across mean pulse rate (x-axis) and amplitude (color). Data are presented as mean ± SE, n=8-10/parameter set.


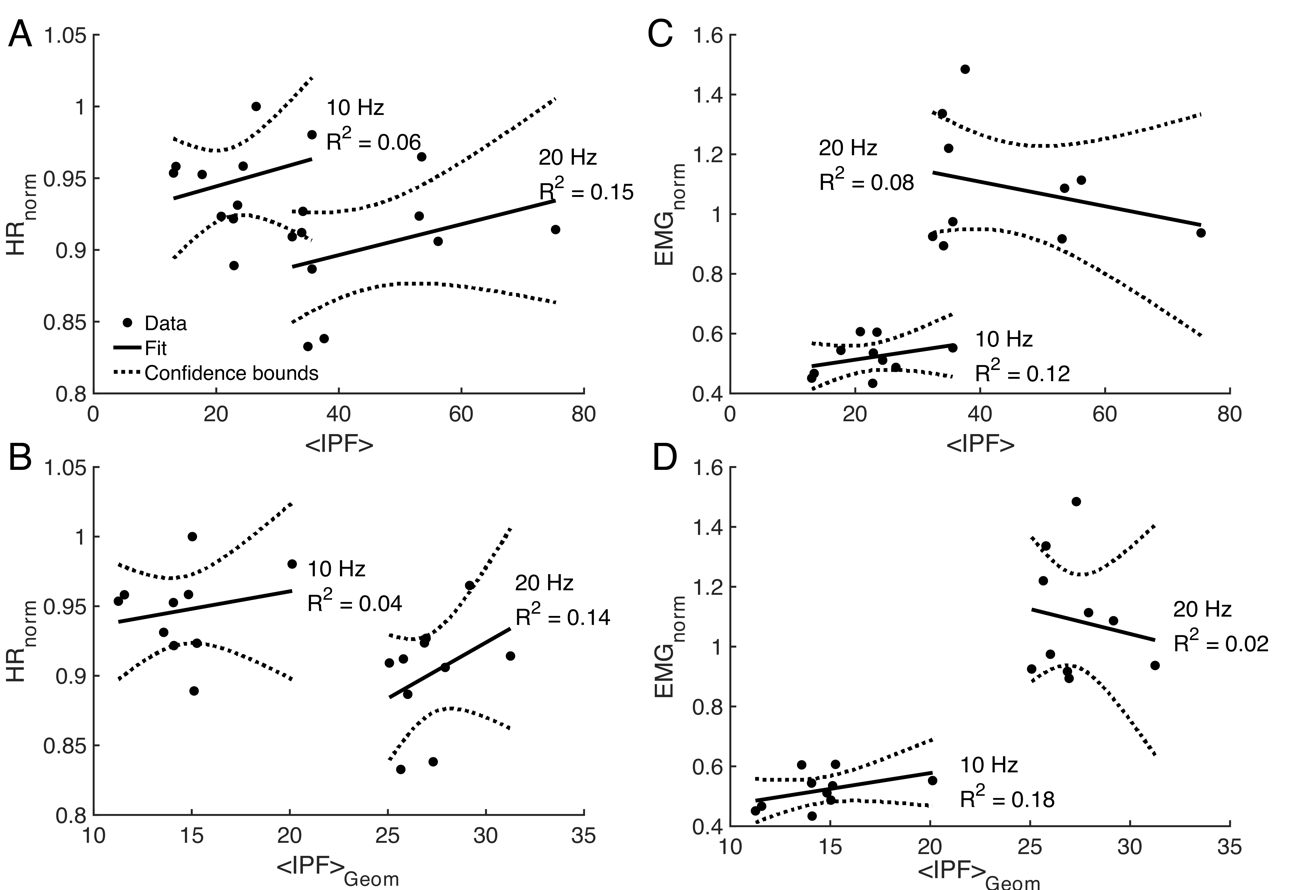


Figure 19. Correlation between in vivo physiological responses and frequency characteristics of random patterns of VNS. A-B) No correlation detected between HR and mean inter-pulse frequency (<IPF>, A) or geometric <IPF> (<IPF>_geo_, B) using linear fits. C-D) No correlation detected between EMG and <IPF> (C) or <IPF>_geo_ (D). Data points are outcomes from individual experiments (n = 10 per MPR value). Coefficient of determination (R^2^) from linear fits. No fit was statistically different from constant values (p > 0.05).


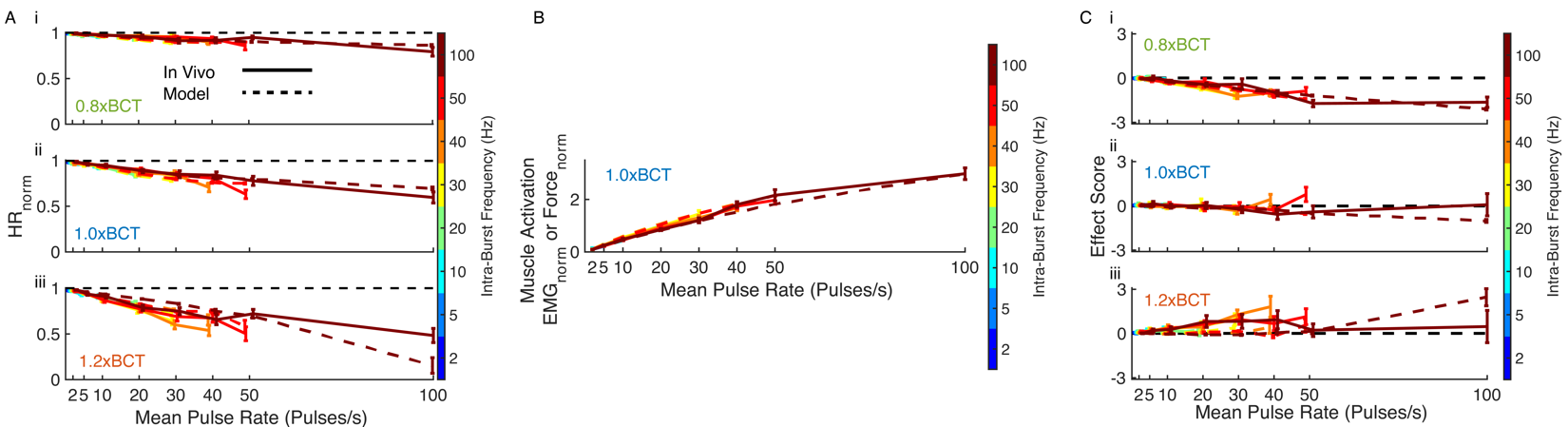


Figure 20. Comparisons of simulated and in vivo data for HRnorm (A), muscle activation (B, EMG_norm_ for in vivo data, Force_norm_ for model data), and effect score (C). Comparison of modeled and in vivo HR_norm_ and effect score performed at 0.8xBCT (i), 1.0xBCT (ii), and 1.2xBCT (iii). EMG_norm_ and Force_norm_ compared for 1.0xBCT. Data are presented as mean ± SE, n=8-10/parameter set for in vivo data, n=10 runs per parameter set for computational models.

Table 7. Error between modeled normalized heart rate (HR_norm, model_) and in vivo HR_norm,in vivo_.

| HR_norm,model_ – HR_norm,in vivo_ | | | Mean Pulse Rate | | | | | | | |
| --- | --- | --- | --- | --- | --- | --- | --- | --- | --- | --- |
|  |  |  | 2 | 5 | 10 | 20 | 30 | 40 | 50 | 100 |
| 0.8xBCT | Burst Frequency (Hz) | 2 | -0.006 |  |  |  |  |  |  |  |
|  |  | 5 | -0.003 | -0.010 |  |  |  |  |  |  |
|  |  | 10 | -0.002 | -0.003 | -0.001 |  |  |  |  |  |
|  |  | 20 | 0.002 | 0.009 | -0.023* | -0.038* |  |  |  |  |
|  |  | 30 | -0.009* | -0.009* | -0.022 | -0.038* | -0.070* |  |  |  |
|  |  | 40 | -0.003 | 0.003 | -0.009 | -0.039 | -0.054* | -0.009 |  |  |
|  |  | 50 | -0.006 | -0.012 | -0.016 | -0.017 | -0.055* | -0.038 | 0.031 |  |
|  |  | 100 | -0.007 | 0.000 | -0.001 | -0.014 | 0.016 | -0.001 | -0.049* | 0.071 |
|  |  |  |  |  |  |  |  |  |  |  |
| 1.0xBCT | Burst Frequency (Hz) | 2 | 0.000 |  |  |  |  |  |  |  |
|  |  | 5 | -0.007 | -0.010 |  |  |  |  |  |  |
|  |  | 10 | 0.000 | -0.014 | -0.007 |  |  |  |  |  |
|  |  | 20 | 0.010 | -0.011 | -0.009 | 0.003 |  |  |  |  |
|  |  | 30 | -0.006 | 0.007 | -0.025 | -0.048 | -0.053 |  |  |  |
|  |  | 40 | 0.011 | -0.012 | -0.018 | -0.019 | -0.045 | 0.036 |  |  |
|  |  | 50 | -0.002 | -0.008 | -0.009 | -0.044 | -0.046 | -0.049 | 0.119 |  |
|  |  | 100 | -0.005 | 0.010 | -0.004 | 0.010 | -0.009 | -0.030 | 0.019 | 0.100 |
|  |  |  |  |  |  |  |  |  |  |  |
| 1.2xBCT | Burst Frequency (Hz) | 2 | -0.003 |  |  |  |  |  |  |  |
|  |  | 5 | -0.010 | 0.000 |  |  |  |  |  |  |
|  |  | 10 | 0.006 | 0.015 | -0.020 |  |  |  |  |  |
|  |  | 20 | 0.002 | 0.002 | -0.001 | -0.028 |  |  |  |  |
|  |  | 30 | -0.005 | 0.006 | 0.025 | 0.054 | 0.076 |  |  |  |
|  |  | 40 | 0.004 | 0.002 | 0.046 | 0.047 | 0.165* | 0.115 |  |  |
|  |  | 50 | 0.008 | 0.015 | 0.047 | 0.054 | 0.057 | 0.078 | 0.081 |  |
|  |  | 100 | -0.003 | 0.026 | 0.028 | 0.097* | 0.080 | 0.095 | -0.024 | -0.328 |
| Model Overestimation | | Model Underestimation | | *Model Error Greater than in vivo SD | | | |  |  |  |

Table 8. Error between modeled normalized force (Force_norm_) and in vivo normalized EMG (EMG_norm_).

| Force_norm_ – EMG_norm_ | | | Mean Pulse Rate | | | | | | | | | | | | | | | |  |
| --- | --- | --- | --- | --- | --- | --- | --- | --- | --- | --- | --- | --- | --- | --- | --- | --- | --- | --- | --- |
|  |  |  | 2 | | 5 | | 10 | | 20 | | 30 | | 40 | | 50 | | 100 | |  |
| 0.8xBCT | Burst Frequency (Hz) | 2 | | -0.006 | |  | |  | |  | |  | |  | |  | |  | |
|  |  | 5 | | -0.015 | | -0.036 | |  | |  | |  | |  | |  | |  | |
|  |  | 10 | | -0.034 | | -0.017 | | -0.069 | |  | |  | |  | |  | |  | |
|  |  | 20 | | 0.000 | | 0.037 | | 0.040 | | 0.000 | |  | |  | |  | |  | |
|  |  | 30 | | 0.016 | | 0.034 | | 0.042 | | 0.075 | | 0.006 | |  | |  | |  | |
|  |  | 40 | | 0.007 | | 0.020 | | 0.078* | | 0.120 | | 0.166 | | 0.163 | |  | |  | |
|  |  | 50 | | 0.014 | | 0.051 | | 0.108* | | 0.153 | | 0.270* | | 0.061 | | 0.172 | |  | |
|  |  | 100 | | 0.014 | | -0.006 | | -0.039 | | -0.023 | | -0.015 | | -0.284 | | -0.342 | | 0.002 | |
| Model Overestimation | | Model Underestimation | | | | *Model Error Greater than in vivo SD | | | | | | | |  | |  | |  | |

Table 9. Error between modeled effect score (Effect Score_model_) and in vivo effect score (Effect Score_in vivo_).

| Effect Score_model_ –  Effect Score_in vivo_ | | | Mean Pulse Rate | | | | | | | |
| --- | --- | --- | --- | --- | --- | --- | --- | --- | --- | --- |
|  |  |  | 2 | 5 | 10 | 20 | 30 | 40 | 50 | 100 |
| 0.8xBCT | Burst Frequency (Hz) | 2 | 0.005 |  |  |  |  |  |  |  |
|  |  | 5 | -0.007 | 0.020 |  |  |  |  |  |  |
|  |  | 10 | -0.020 | -0.069 | -0.035 |  |  |  |  |  |
|  |  | 20 | -0.080 | -0.211 | -0.005 | 0.009 |  |  |  |  |
|  |  | 30 | 0.069 | -0.036 | -0.005 | 0.159 | 0.426 |  |  |  |
|  |  | 40 | -0.045 | -0.149* | -0.147 | -0.001 | 0.407 | -0.125 |  |  |
|  |  | 50 | -0.016 | 0.019 | -0.029 | -0.375 | -0.077 | -0.082 | -0.544 |  |
|  |  | 100 | 0.007 | -0.154 | -0.087 | -0.026 | -0.326 | 0.035 | 0.538 | -0.474 |
|  |  |  |  |  |  |  |  |  |  |  |
| 1.0xBCT | Burst Frequency (Hz) | 2 | -0.004 |  |  |  |  |  |  |  |
|  |  | 5 | 0.034 | 0.048 |  |  |  |  |  |  |
|  |  | 10 | 0.023 | 0.066 | 0.112 |  |  |  |  |  |
|  |  | 20 | -0.098 | -0.021 | -0.095 | 0.000 |  |  |  |  |
|  |  | 30 | 0.028 | -0.140 | 0.033 | -0.091 | 0.202 |  |  |  |
|  |  | 40 | -0.045 | 0.013 | -0.053 | -0.071 | 0.137 | -0.607 |  |  |
|  |  | 50 | -0.013 | -0.062 | -0.164 | -0.059 | -0.122 | 0.055 | -1.310 |  |
|  |  | 100 | -0.024 | -0.198 | -0.043 | -0.177 | 0.086 | 0.292 | -0.098 | -1.098 |
|  |  |  |  |  |  |  |  |  |  |  |
| 1.2xBCT | Burst Frequency (Hz) | 2 | 0.052 |  |  |  |  |  |  |  |
|  |  | 5 | 0.076 | 0.008 |  |  |  |  |  |  |
|  |  | 10 | -0.057 | -0.117 | 0.008 |  |  |  |  |  |
|  |  | 20 | -0.074 | -0.094 | -0.162 | 0.110 |  |  |  |  |
|  |  | 30 | 0.000 | -0.210 | -0.279 | -0.381 | -0.595 |  |  |  |
|  |  | 40 | -0.052 | -0.166 | -0.450 | -0.384 | -1.245* | -1.420 |  |  |
|  |  | 50 | -0.075 | -0.170 | -0.349 | -0.581 | -0.783 | -0.880 | -0.598 |  |
|  |  | 100 | 0.008 | -0.203 | -0.309 | -0.894 | -0.914 | -0.858 | -0.094 | 1.986 |
| Model Overestimation | | Model Underestimation | | *Model Error Greater than in vivo SD | | | |  |  |  |


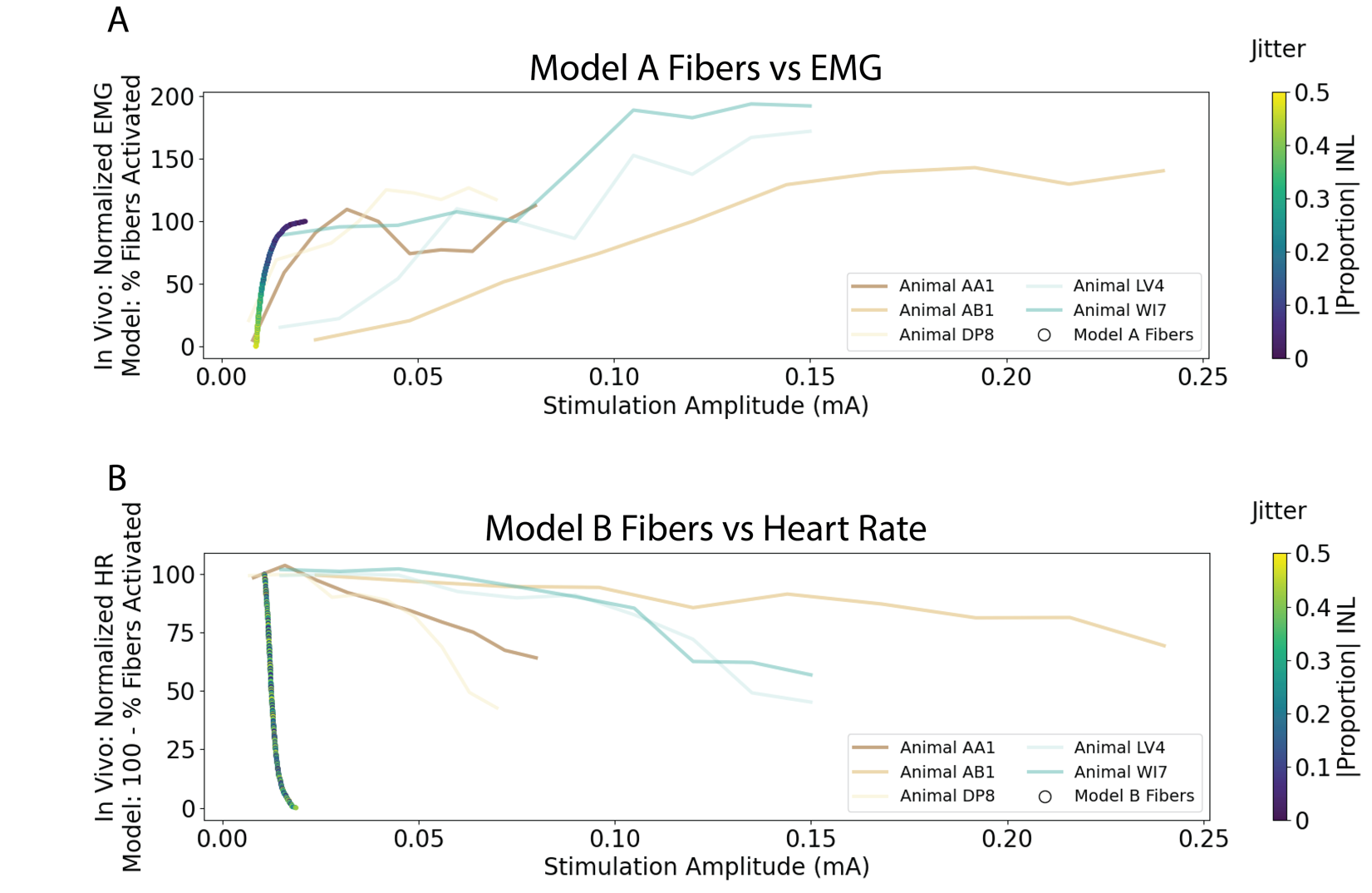


Figure 21. Recruitment curves for modeled A and B fibers colored to indicate fiber “jitter” (i.e., longitudinal shift of fibers as a proportion of internodal length) overlaid with in vivo EMG (A) and normalized heart rate (B) responses in five animals across stimulation amplitudes.


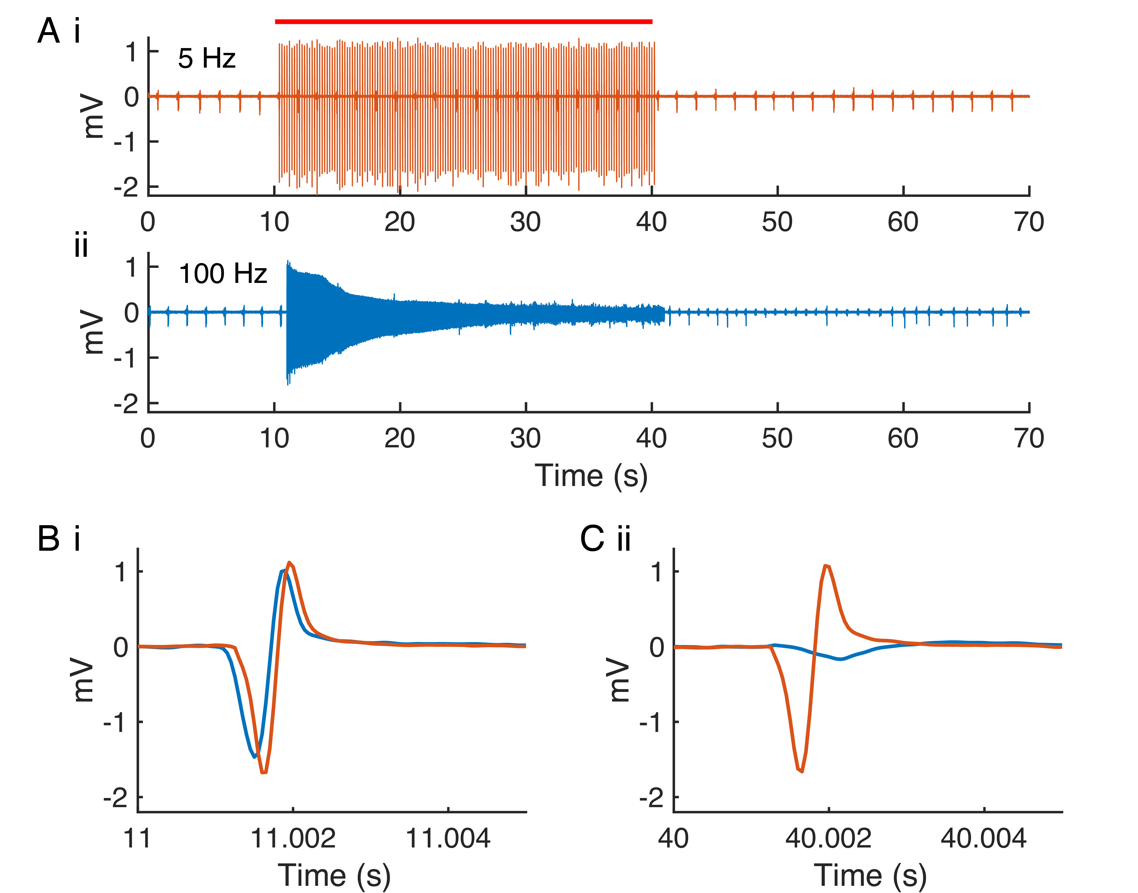


Figure 22. EMG waveform depression during high-frequency VNS. A) EMG recordings of two trials within the same animal (XD0). EMG waveform amplitude stays consistent during 5 Hz stimulation B). Evoked EMG waveform amplitude is similar at beginning of the trial (i) and is greatly reduced at the end of the trial during 100 Hz stimulation.


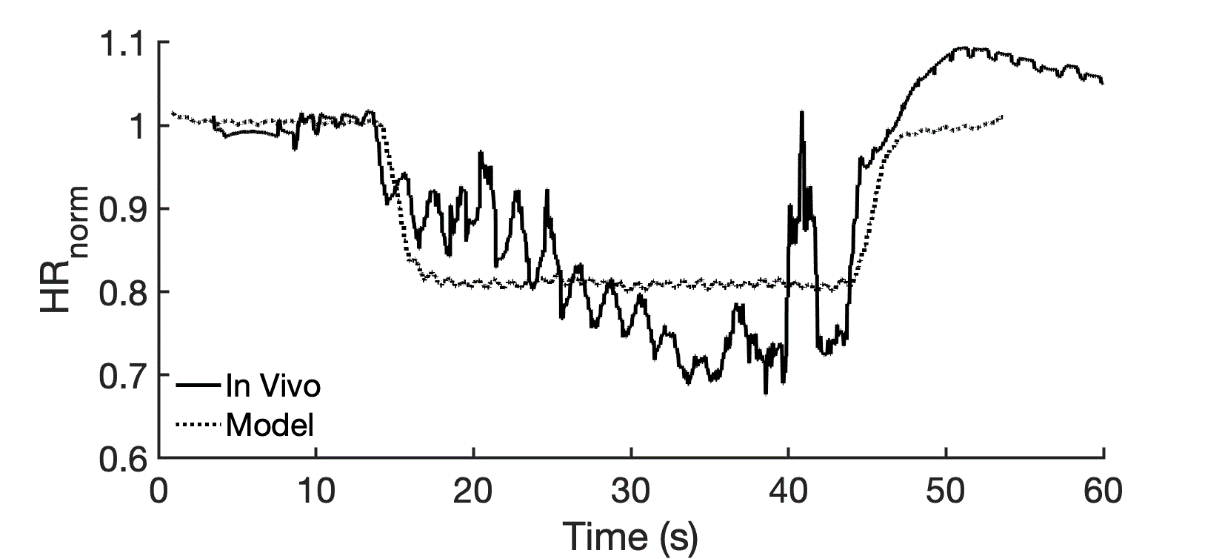


Figure 23. Comparison of dynamic normalized heart rate (HR_norm_) values for representative in vivo trial (animal HU0, solid line) and simulation (dashed line) during 1.0xBCT, 100 Hz intra-burst frequency, and 50 MPR stimulation.
